# Docking Simulations of G-Protein Coupled Receptors Uncover Crossover Binding Patterns of Diverse Ligands to Angiotensin, Alpha-Adrenergic and Opioid Receptors: Implications for Cardiovascular Disease and Addiction

**DOI:** 10.3390/biom15060855

**Published:** 2025-06-11

**Authors:** Harry Ridgway, Graham J. Moore, Laura Kate Gadanec, John M. Matsoukas

**Affiliations:** 1 Institute for Sustainable Industries and Liveable Cities, Victoria University, Melbourne, VIC 8001, Australia; ridgway@vtc.net; 2 THERAmolecular, LLC, Rodeo, NM 88056, USA; 3 Pepmetics Inc., 772 Murphy Place, Victoria, BC V8Y 3H4, Canada; mooregj@shaw.ca; 4 Immunology and Translational Research Group, Institute for Health and Sport, Victoria University, Melbourne, VIC 3030, Australia; 5 Department of Physiology and Pharmacology, Cumming School of Medicine, University of Calgary, Calgary, AB T2N 1N4, Canada; 6 NewDrug/NeoFar PC, Patras Science Park, 26504 Patras, Greece; 7 Department of Chemistry, University of Patras, 26504 Patras, Greece

**Keywords:** alpha-adrenergic receptor, angiotensin II type 1 receptor, angiotensin receptor blockers, bisartans, computer-aided docking simulations, G-protein-coupled receptors, opioid receptors

## Abstract

Recent bioassay studies have unexpectedly supported the high (computationally predicted) binding affinities of angiotensin receptor blockers (ARBs) at α-adrenergic receptors (αARs) in isolated smooth muscle. Computational predictions from ligand docking studies are consistent with very low concentrations of ARBs (e.g., sartans or bisartans) that partially reduce (20–50%) the contractile response to phenylephrine, suggesting that some ARBs may function as partial inverse agonists at αARs. Virtual ligand screening (docking) and molecular dynamics (MD) simulations were carried out to explore the binding affinities and stabilities of selected non-peptide ligands (e.g., ARBs and small-molecule opioids) for several G-protein coupled receptor (GPCR) types, including angiotensin II (AngII) type 1 receptor (AT_1_R), α1AR, α2AR, and μ-(µOR) and ժ-opioid receptors (ժOR). Results: All ligands docked preferentially to the binding pocket on the cell surface domain of the GPCR types investigated. Drug binding was characterized by weak interactions (hydrophobic, hydrogen bonding, pi-pi) and stronger ionic and salt-bridge interactions (cation-pi and cation-anion interactions). Ligands specific to each GPCR category showed considerable cross-binding with alternative GPCRs, with small-molecule medications appearing less selective than their peptide or ARB functional equivalents. ARBs that exhibit higher affinities for AT_1_R also demonstrate higher affinities for µORs and ժORs than opiate ligands, such as fentanyl and naltrexone. Moreover, ARBs had a higher affinity for αARs than either alpha agonists (epinephrine and phenylephrine) or inhibitors (prazosin and doxazosin). MD simulations of membrane-embedded ARB-GPCR complexes proved stable over nanosecond time scales and suggested that some ARBs may behave as agonists or antagonists depending on the GPCR type. Based on the results presented in this and related investigations, we propose that agonists bind to the resting A-site of GPCRs, while inverse agonists occupy the desensitizing D-site, which partial agonists like morphine and fentanyl share, contributing to addiction. ARBs block both AngII and alpha receptors, suggesting that they are more potent antihypertensive drugs than ACE inhibitors. ARBs have the potential to inhibit morphine tolerance and appear to disrupt receptor desensitization processes, potentially by competing at the D-site. Our results suggest the possible therapeutic potential of ARBs in treating methamphetamine and opiate addictions.

## 1. Introduction

Angiotensin receptor blockers (ARBs), such as sartans, have long been established for their efficacy in managing hypertension and cardiovascular diseases [1,2]. Their mechanism of action is by selectively blocking the angiotensin II (AngII) type 1 receptor (AT_1_R) and inhibiting the vasoconstrictive and aldosterone-secreting effects of AngII [3]. This action aids in decreasing blood pressure and reducing cardiovascular strain, making ARBs invaluable in the treatment of conditions such as hypertension, heart failure, and chronic kidney disease [4,5,6]. In addition to their main uses, ARBs are known for their favorable safety profiles, particularly when compared with other anti-hypertensive medications. They are especially beneficial for individuals who experience adverse effects from other cardioprotective medications, such as angiotensin-converting enzyme (ACE) inhibitors [7]. Unlike ACE inhibitors, which may cause side effects like cough or angioedema due to their impact on bradykinin, ARBS do not typically cause these issues because they block the AT_1_R rather than affecting bradykinin metabolism. This makes ARBs a well-tolerated alternative for managing hypertension in patients who cannot tolerate ACE inhibitors while still effectively controlling blood pressure and providing cardiovascular protection [4,8]. ARBs also protect against vascular damage, reduce diabetic nephropathy, and decrease the risk of stroke [9]. The emergence of second-generation derivatives, like bisartans, which feature two biphenyl tetrazole groups, has broadened their therapeutic potential to include combating respiratory viral infections, such as severe acute respiratory syndrome coronavirus 2 (SARS-CoV-2), influenza, and respiratory syncytial virus (RSV) [10,11]. Tetrazoles are unnatural 5-member heterocyclic compounds consisting of 4-nitrogen and 1-carbon atoms and have been shown to exhibit diverse pharmacological properties against several diseases [12], including cancer [13], anti-bacterial [14], anti-viral [15], anti-depressant [16], and anti-inflammatory [17] properties.

The AT_1_R, alpha 1 (α1AR), alpha 2 adrenergic receptors (α2AR), and mu-opioid receptor (µOR) play significant roles in addiction. These receptors are involved in mediating the reinforcing effects of addictive substances and the development of tolerance and dependence. Understanding their interactions and modulation by substances like ARBs and other ligands is crucial for developing targeted therapies for addiction treatment [18,19]. Our laboratory’s previous studies focused on evaluating the effects of commercially available sartans (like candesartan and telmisartan) and bisartans in isolated arteries from New Zealand White rabbits [20]. These studies demonstrated that bisartans have the ability to reduce or desensitize the contractile response to the α1AR agonist phenylephrine, with the exception of benzimidazole-N-biphenyl tetrazole (ACC519T), which induces an up-sensitization /re-sensitization effect on the α1AR [21]. This ability to regulate receptor sensitivity and decrease tolerance, dependence, and withdrawal suggests a promising approach to addiction treatment [22].

Motivated by these findings [21], we conducted a comprehensive analysis of the binding energies of several ARBs with ligands specific to key receptors: AT_1_R, α1AR, α2AR, and µOR. Using advanced computer-aided docking (CAD) simulations, we determined the interactions between these compounds and their respective G-protein-coupled receptors (GPCRs). This computational approach enabled us to understand how ARBs bind to and interact with these receptors, shedding light on their influence on receptor function in diverse physiological contexts. We aimed to gain molecular insights into the mechanisms through which ARBs modulate the receptor signaling pathways. Such insights are crucial for understanding the pharmacological effects of these compounds on cardiovascular regulation, stress responses, and pain modulation. Additionally, by evaluating these molecular mechanisms, our research contributes to the potential development of targeted pharmacotherapies that could enhance treatment outcomes for various disorders, including addiction and cardiovascular disease. Further studies are essential to elucidate the specific mechanisms by which ARBs, including potential compounds like ACC519T, affect α1AR sensitivity and their broader implications for addiction treatment. This ongoing research could pave the way for novel therapeutic strategies that leverage our understanding of GPCR interactions to improve patient care and quality of life.

## 2. Methods

### 2.1. Ligand Docking Protocols

Ligand docking to GPCR binding sites was performed using AutoDock VINA with default settings [23]. The setup was implemented using the YASARA molecular modeling program [24], and the best hit observed from a minimum of 500 runs per ligand expressed as the kcal/mol of the “free energy of binding” (FEB) is given in the figures. Note that all FEBs are reported as positive values (more positive = stronger binding). Ligand dissociation constants (K_d_) were expressed as the logarithm of pM values (Log_10_K_d_). Global docking of individual ligands was initiated by introducing the ligand into a random location inside a walled cell, enclosing the entire GPCR with a random ligand conformation, using a minimum of 900 runs per ligand. The globally docked ligands clustered around specific preferred receptor-binding sites. Two complexes belonged to different clusters if the ligand RMSD was greater than 5.0 Å. The lowest-energy conformation from each cluster is reported.

### 2.2. MD Simulations of Ligand-Receptor Complex Stabilities

Molecular dynamics (MD) simulations were performed using the scripted protocols provided by YASARA [24]. The molecular system (e.g., a ligand-receptor complex) was positioned in cubic periodic boundaries with all atoms more than 10 Å away from any boundary wall. The protocol included an initial optimization of the hydrogen bonding network [25] to increase protein stability, and the invocation of a pKa prediction model to fine-tune the residue protonation states at a pH of 7.4 [26]. For peptide docking (e.g., deltorphin or AngII), residue sidechain rotations were fixed, while the backbone atoms remained fully flexible. This restraint was necessary since the inclusion of residue flexibility exceeded the maximum dihedrals that VINA could accurately compute. Where possible, the fidelity of VINA was tested by comparing x-ray crystallographic ligand conformations against docked drug poses. Na^+^ and Cl^−^ ions were introduced at a physiological concentration of 0.9 wt%, with an excess of either ion to neutralize the cell. After the steepest descent and simulated annealing minimizations to remove clashes, the simulation was run for 155–200 nanoseconds (ns) using the AMBER14 force field [27] for the solute, GAFF2 [28] and AM1BCC [29] for ligands and TIP3P for water. All simulations were performed using an NVIDIA 4090 GPU graphics card that handled the pair interatomic interactions for the solvent electrostatics and Lennard-Jones forces. The cutoff was 16 Å for Van der Waals forces (the default value used by AMBER [30]. No cutoff was applied to the electrostatic forces (using the Particle Mesh Ewald algorithm [31]). The equations of motion were integrated using multiple timesteps: 1.25 femtoseconds (fs) for “normal” simulations, 2.5 fs for “fast” simulations for bonded interactions, and 2.5 fs (normal) or 5.0 fs (fast) for non-bonded interactions at a temperature of 310 K and a pressure of 1.0 atm (NPT ensemble) using algorithms described in detail previously [32].

### 2.3. MD Simulations of GPCR-Membrane Complexes

MD simulations of membrane-embedded GPCRs were automated using a modification of the scripted algorithm supplied in YASARA Structure (www.yasar.org; accessed 10 December 20204). The source code for this simulation protocol and associated visualizations of the individual steps may be found at http://www.yasara.org/membranemd (accessed on 28 February 2025). The algorithm was launched by initially scanning the GPCR for exposed transmembrane (TM) helices. The criteria for TM helix identification were as follows: (i) helices in excess of sixteen residues; (ii) each helix with more than seven hydrophobic residues; and (iii) more than three exposed residues with accessible sidechain surface areas > 30% of the maximum. The major axis vectors of these helices (i.e., the direction vectors of the least-squares lines through the Calpha atoms) were summed to obtain the major axis of the protein. The receptor was then oriented parallel to the Y-axis and orthogonal to the XZ plane. The lipid bilayer membrane model was positioned along the major axis by scanning the TM region of the GPCR for the largest number of exposed hydrophobic residues (see definition above) and a width of 28 Å, corresponding to the membrane core. Following the placement of an equilibrated membrane structure comprising phosphatidyl−ethanolamine molecules at this location (designated as “MemCenterY”), the system was enclosed in periodic boundaries with dimensions of 86 × 350 × 86 Å. The GPCR was temporarily scaled by a factor of 0.9 along the XZ-axes, followed by the removal of strongly clashing membrane lipids, i.e., lipids with an atom closer than 0.75 Å to a protein atom. Temporary protein scaling, which was needed to avoid the deletion of too many lipids around the protein, was then slowly removed during a short simulation at 298 °K in vacuo. The protein, with all atoms fixed, was scaled by 1.02 along the XZ-axes every 200 femtoseconds (fs), while the membrane was allowed to move but restrained to ideal geometry by pulling lipid residues with an atom further than 21.5 Å away from MemCenterY back into the membrane and by pushing outwards phosphorus atoms closer than 14 Å to MemCenterY. The force field used was AMBER14 [27], with Lipid17/GAFF2/AM1BCC parameters applied for non-standard residues [28,33,34]. As soon as the protein reached its original size, the protein sidechain pKas were calculated [35], protonation states were assigned according to pH 7.4, and the simulation cell was filled with TIP3P water, 0.9 wt% NaCl, and counter ions [26]. The main simulation was then run with Particle Mesh Ewald [31] long-range electrostatics and 12.0 Å cutoff for non-bonded real space forces, a 5.0 fs time-step with constrained hydrogen atoms, and at constant pressure (1.0 atm) and temperature (310 °K; NPT ensemble), with thermostat and barostat coupling to time-average values as described previously [32]. The pressure values calculated along the X- and Z-axes (i.e., parallel to the membrane plane) were averaged before re-scaling the cell, ensuring that the membrane aspect ratio remained fixed (semi-isotropic approach). During the initial 250 picoseconds (ps) equilibration period, the membrane was restrained to avoid distortions, while the simulation cell adapted to the pressure exerted by the membrane (see above; additionally, water molecules that got closer than 14 Å to MemCenterY were pushed outside).

## 3. Results

### 3.1. Primary Binding Site for GPCRs

Ligand binding for FDA-approved and experimental drugs was examined across three GPCRs: AT1R, αAR, and µOR. No structural homology exists among the three extracellular binding pockets, and each receptor-ligand interacts with a distinct set of amino acids. For each receptor studied, all ligands were found to predominantly bind in the binding pocket at the cell surface domain, where natural agonists are usually located. However, global mapping of αARs revealed two lower-affinity allosteric sites and an allosteric cleft appearing between receptor dimer structures, which seems to recognize ARBs and alpha-blockers but not alpha agonists. Interestingly, bisartans were found to bind to the αAR and µOR pockets with minimal or no strong ionic interactions with tetrazolate anions (e.g., tetrazolate—arginine interactions). Additionally, the binding energies appear to be derived from cumulative weaker interactions, such as H-bonds, dipolar, quadrupolar, and hydrophobic interactions. This may be because, unlike AT_1_Rs, there is a paucity of polar amino acid sidechains present in the binding pockets of both αARs and µORs; however, access to polar (salt-bridge) interactions may occur as a result of cooperative agonist activation of receptors that are not visible in these CAD studies, and the transition of receptors from low-affinity state to high-affinity state requires energy, which could be provided by salt-bridging [36].

#### 3.1.1. Ligand Docking at AT_1_R

**Figure 1:**
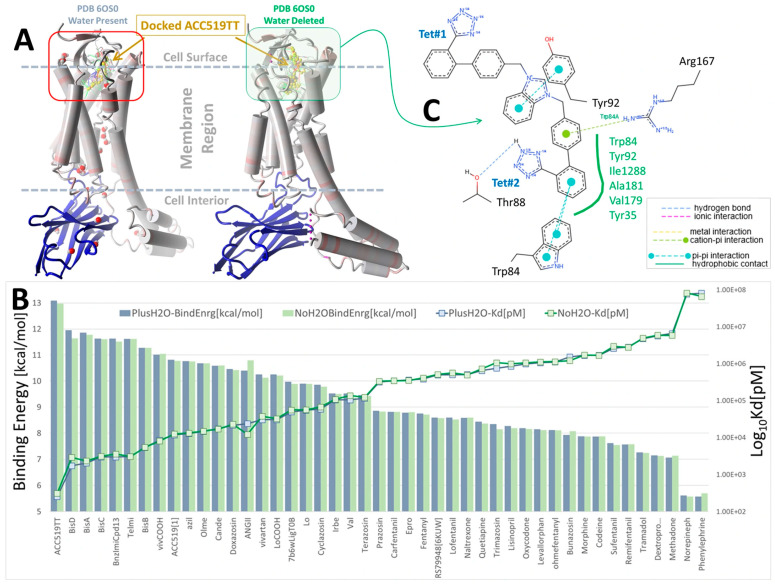
Docking of selected ARBs (FDA-approved sartans and experimental bisartans) and opioid drugs (fentanyl, naltrexone, morphine and oxycodone) to synthetic NB-stabilized AT_1_R (PDB: 6OS0) (https://www.rcsb.org/structure/6os0, accessed on 4 January 2025). Docking was performed in the presence and absence of co-crystallized water using AutoDock VINA with 995 runs per ligand (see Methods section). (**A**) Structure of AT_1_R (6OS0) showing the approximate docking region (red box) at the cell surface domain and the bound synthetic NB (blue ribbons). (**B**) Docking energies (kcal/mol) and logarithm of the calculated ligand dissociation constant, Kd (pM). Blue bars = docking with co-crystallized water present, while green bars = docking with deleted water, indicating that bound water had little influence on ligand-receptor interactions. (**C**) 2D interaction diagram of ACC519TT docked into the water-free pocket of 6OS0 (https://proteins.plus/; accessed on 13 April 2024). Ligand binding was driven primarily by hydrophobic interactions with multiple receptor residues (green solid line; green residue labels), a cation-pi interaction between Arg^167^ and the phenol group of ACC519TT located proximate to the central imidazole (green dashed line), and pi-pi interactions between Trp^84^ and the phenol group adjacent to anionic tetrazole#2 (cyan dashed lines).

The pharmacological properties of various ARBs at AT_1_R are well understood, and an investigation of CAD for these compounds can provide a baseline analysis and potential validation of CAD methods. Figure 1 illustrates that the most potent ARBs, namely bisartans, generally exhibit the highest binding energies/affinities, followed by various sartans (although the relatively low affinity of telmisartan is a notable anomaly). Interestingly, alpha-blockers like doxazosin also demonstrate a substantial affinity for AT_1_R, perhaps illustrating reciprocal modulation of AT_1_R by alpha ligands and alpha receptors by ARBs, although the former has yet to be proven by bioassay. In addition, opiates like carfentanyl and fentanyl show unexpectedly high affinities for AT_1_R, yet to be verified by bioassay; however, ligand binding does not necessarily imply bioactivity.

**Figure 2:**
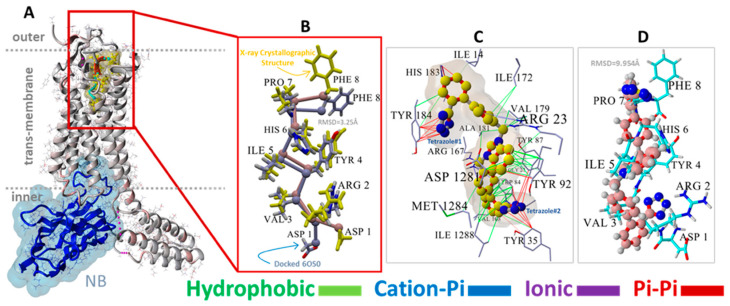
(**A**) Structure of NB-stabilized (blue ribbons, water-accessible surface shading) AT_1_R (6OS0) with docked native AngII (yellow molecule). (**B**) Magnified view showing the superimposition of VINA-docked AngII (blue carbon atoms) against the 6OS0 X-ray crystallographic pose of AngII (maroon carbon atoms with yellow side chains). Due to VINA limitations (see Section 2), AngII docking was performed by retaining the full torsional flexibility of the backbone carbon atoms but disallowing sidechain rotations. Using this simplification, VINA was able to predict a reasonable pose for AngII with a root-mean-square deviation (RMSD) of 3.25 Angstrom (Å) for the overlapped structures. The most significant departure from the X-ray pose involved the terminal Phe^8^ and Asp^1^ groups. As shown in the image, the superimposition of the remaining AngII residues was quite close, validating the efficacy of the VINA docking algorithm. (**C**) Principal residue interactions of the VINA-docked experimental bisartan ACC519TT indicating that both anionic tetrazole groups entered pi-pi interactions (red lines) with proximal aromatic residues, including Tyr^35^, Tyr^92^, Tyr^184^, and Trp^84^. The central cationic imidazole group also underwent pi-pi bonding with Tyr^92^, except for one phenyl group of ACC519TT entering a weak cation-pi interaction with Arg^23^ (light blue to gray lines). All other interactions were hydrophobic (green lines). (**D**) Orientation of VINA-docked bisartan (maroon carbon atoms, ball-cylinder rendering) reflected that of AngII (cyan carbon atoms, tube rendering) and was approximately parallel to the long axis of the receptor.

For AT_1_R, the acid groups of sartans were anticipated to interact with nearby R167 and possibly K199 in the crystal structure [37,38]; however, these interactions were not evident (Figure 2). Surprisingly, in Figure 2, the negatively charged tetrazole of ACC519TT, located near R167, does not interact with the positively charged guanidino side chain (energy = 20–50 kcal/mol), suggesting that a series of smaller interactions may compensate for this missing large energy, or that there could be significant geometric constraints on binding. However, subsequent molecular dynamics simulations showed that this interaction occurs over time, highlighting a potential limitation of CAD studies, in which only the first step of the binding interaction is observed.

#### 3.1.2. Ligand Docking at α1AR and α2AR

**Figure 3:**
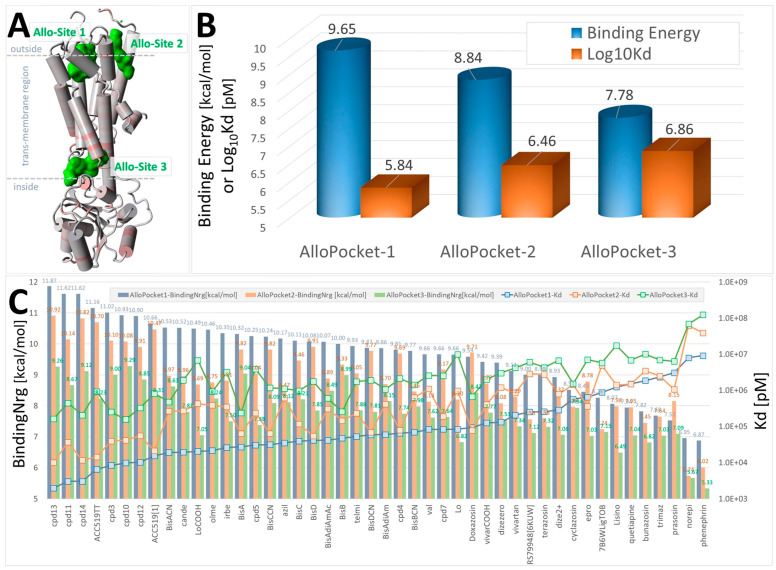
Docking of selected FDA-approved sartans (e.g., candesartan), experimental sartans (e.g., ACC519TT), and opioid drugs (e.g., prazosin) to the α2CAR GPCR, PDB 6KUW. The ligand structures are shown in Supplementary Materials, Figure S1. Ligands were docked using AutoDock VINA to the three best allosteric pockets predicted by the Protein Allosteric Sites Server (PASSer) at https://passer.smu.edu/ (accessed on 4 April 2024). Allosteric pockets were identified using the Ensemble Model at PASSer. (**A**) 6KUW is rendered in cartoon format with alpha cylinders colored according to residue charge (red = more negative). Green shading = locations of the top three predicted allosteric sites (Allo-Site 1, 2 and 3). The predicted allostery probabilities for the three sites were 80.992, 70.797, and 28.466, respectively [39]. (**B**) Average ligand binding energies (blue cylinders) and means of the calculated dissociation constants (Log_10_Kd; orange bars) for all drugs. Allo-Site 1 was generally favored by most drugs tested, consistent with the allostery prediction probabilities. (**C**) Docking results for each drug tested across the three allosteric sites showed that the five drugs with the highest (most positive) docking scores were the experimental sartans cpd13, cpd11, cpd14, cpd3, and ACC519TT.

**Figure 4:**
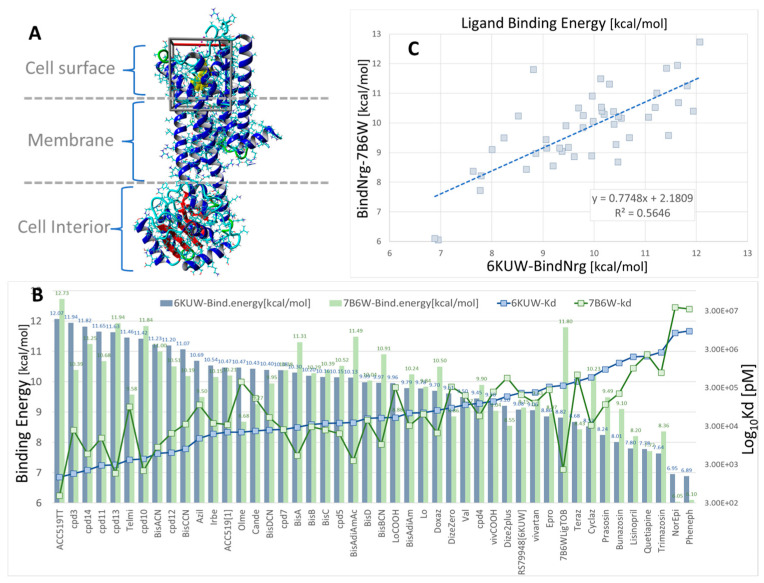
Docking of 47 selected opiates and sartans against human α1BAR, α2CAR, and GPCRs. The ligand structures are shown in Supplementary Materials Figure S1. (**A**) X-ray crystallographic structure of human α1BAR (PDB: 7B6W) in complex with the inverse agonist (+)-cyclazosin (yellow spheres). The docking region of interest is indicated by a 3D box (walled periodic boundaries). The structure of α2CAR (PDB: 6KUW) appears very similar but is not shown here. (**B**) Binding energies and calculated Kd values for all 47 ligands docked to 6KUW and 7B6W. The experimental bisartan, ACC519TT, exhibited the strongest binding affinity for both GPCR types. In general, the opiates and sartans docked similarly (in terms of binding energies) to both GPCRs. One notable exception was ligand 7B6WLigTOB, which was designed specifically for the 7B6W receptor (https://www.rcsb.org/structure/7B6W; accessed on 4 January 2025). (**C**) Scatter plot showing the correlation between 7B6W and 6KUW binding energies (R^2^ = 0.5646, *n* = 47).

**Figure 5:**
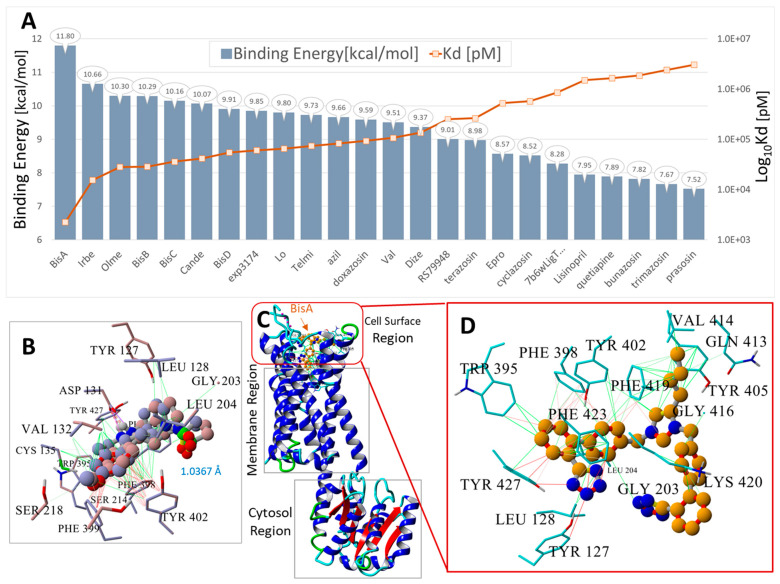
Docking of 24 FDA-approved and experimental sartans and selected opiates to human α2CAR GPCR, PDW: 6KUW. The ligand structures are shown in Supplementary Materials Figure S1. (**A**) Blue bars = binding energies (kcal/mol); orange line and markers = logarithm of the calculated dissociation constant (Log_10_Kd). (**B**) Superimposition of the 6KUW X-ray pose of compound RS79948 on the same VINA-docked ligand. The RMSD of the superimposed molecules was 1.5462 Å, indicating that VINA was able to predict a reasonable pose for this molecule. (**C**) Structure of 6KUW showing bisartan BisA docked into the cell surface domain of 6KUW. BisA was oriented roughly parallel to the long axis of the 7TM region of the receptor. (**D**) Residue interactions of docked BisA in the cell surface pocket of 6KUW. The dominant interactions include hydrophobic bonding (green lines) between the butyl group of BisA and residues Phe^419^, Val^414^, and Gln^413^; pi-pi interactions (magenta to red lines) between Phe^419^ and the BisA imidazole group; and pi-pi interactions between Trp^395^, Phe^398^, Tyr^402^, and Tyr^427^ and the BisA biphenyl groups. The anionic tetrazole group near Gly^203^ did not strongly interact but was proximal to the cationic Lys^420^ residue and probably exhibited a weak ionic interaction with this residue.

**Figure 6:**
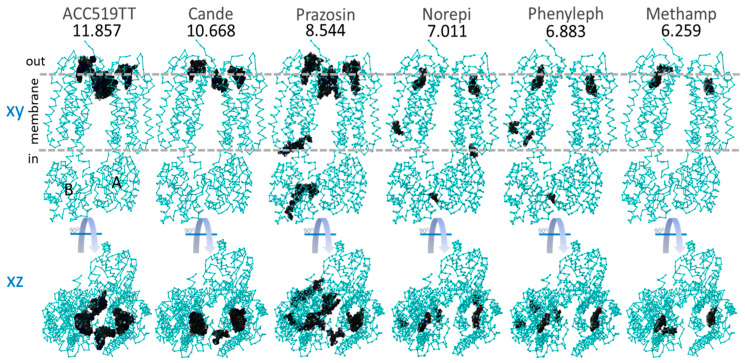
Global docking of six selected sartans and opiate drugs to the dimeric form of α2CAR (PDB: 6KUW). Docking was performed using AutoDock VINA with 990 runs for each ligand (see Methods). The numbers below the drug names are the strongest observed binding energies from all 990 runs. **Top row**: XY-plane projections showing areas (dark patches) where drug binding occurred. A = A chain and B = B chain. Horizontal dashed lines indicate the approximate inner and outer boundaries of the cytoplasmic membrane. **Bottom row**: XZ-plane projection from the top row, also showing areas where drug binding was observed. The binding of both sartans (ACC519TT and candesartan) and methamphetamine were restricted to the cell surface allosteric pocket.

As outlined above, the effects of ARBs on alpha-adrenergic receptors were investigated to determine whether the effects of ARBs were mediated by receptor binding or by interference with intracellular signaling pathways leading to a contractile response. The availability of crystal structures for the alpha receptor in public archives allowed an investigation of the alpha receptor in monomer (Figure 3, Figure 4 and Figure 5) and dimer (Figure 6) forms, with the latter including co-crystallized G protein. Unexpectedly high affinities were observed for bisartans and some sartans at the alpha receptors, more than for alpha-blockers (Figure 3, Figure 4 and Figure 5). Although ARBs block vasoconstriction induced by alpha agonists, this effect is not greater than that of alpha-blockers, implying that the binding affinities observed by CAD do not necessarily reflect biopotency. Figure 6 shows that the global docking of bisartan (ACC519TT) and sartan (candesartan) is of higher affinity than that of the alpha-blocker prazosin as well as for several alpha agonists (norepinephrine, phenylephrine, and methamphetamine), which could imply that CAD data are unreliable (computer bias or error) or that ligand binding can occur without necessarily causing bioactivity.

#### 3.1.3. Ligand Docking at µOR and δOR

**Figure 7:**
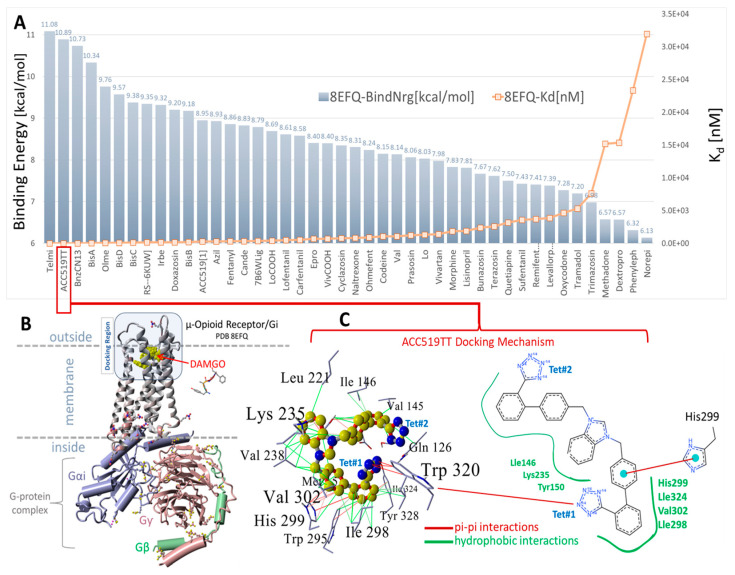
(**A**) Docking results for selected opiates and FDA-approved and experimental sartans to the cell surface domain of the µOR, PDB: 8FEQ. Telmisartan and experimental ACC519TT exhibited the first (11.08 kcal/mol) and second strongest (10.98 kcal/mol) docking energies, respectively. The next strongest binding opiate drugs were RS79948 (9.35 kcal/mol) and doxazosin (9.20 kcal/mol). (**B**) Structure of PDB 8EFQ and the associated cytosolic G-protein complex. A single Arg^213^ residue was situated on the outer loop region of the cell surface docking domain of 8EFQ (rendered as balls in (**B**)). (**C**) Docking mechanism of ACC519TT in the cell surface domain of 8EFQ. The left panel of (**C**) shows a 3D representation of the ligand-receptor interactions, while the right panel shows a 2D interaction diagram (https://proteins.plus/; accessed on 28 February 2025). ACC519TT and other bisartans were found to bind to the αAR (Figure 3, Figure 4, Figure 5, Figure 6 and Figure 7) pockets with minimal or no ionic (salt-bridge) interactions with the tetrazolate anions. In the case of 8EFQ, this was not surprising since only one arginine residue (Arg^213^) was associated with the docking domain outer loop. However, several bisartans (BisA, BisB, BisC, and BisD) exhibited weaker pi-pi interactions with the singly protonated His^299^. Bisartan ACC519TT adopted a bent ‘horseshoe’ conformation, which was stabilized by pi-pi resonance interactions (red lines) between tetrazole group #1 (Tet#1) and Trp^320^. The central cationic imidazole group was bound through hydrophobic interactions with Tyr^150^, Lys^235^, and Val^238^. The penultimate phenyl group (proximal to Tet#1) established hydrophobic and pi-pi interactions with residues Ile^324^, Tyr^328^, Trp^925^, and His^299^. Several additional hydrophobic interactions also contributed to drug binding to the receptor involving 8EFQ residues Tyr^150^, Leu^221^, Lys^235^, Val^238^, Val^145^, Val^302^, Gly^327^, Ile^146^, Ile^298^, Met^153^, and Thr^122^.

**Figure 8:**
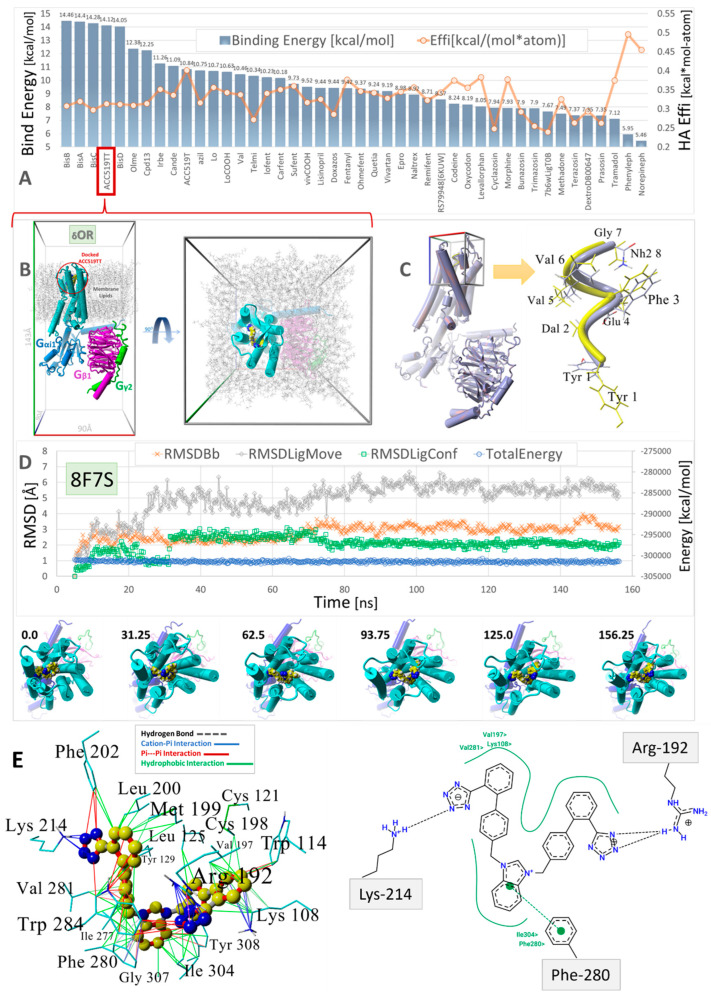
Virtual ligand screening of a range of approved and experimental anti-hypertensive sartan drugs and opioids to the human GPCR δ-opioid receptor (δOR, PDB: 8F7S). (**A**) Ligands were docked using AutoDock with 900 runs per ligand (see the Section 2). To account for the influence of molecular size on the binding energies, the binding efficiencies (“Effi”; kcal/mol-atom) were also computed by dividing the binding energies by the number of heavy atoms per ligand (orange line). (**B**) Molecular system (viewed down the Z and Y axes) used to evaluate the stability of docked bisartan ACC519TT in NPT equilibrium MD simulations (see Methods). The drug-receptor complex was embedded in a lipid bilayer membrane comprising phosphatidyl−ethanolamine (1-palmitoyl, 2-oleoyl) with explicit TIP3P solvation and NaCl counterions (water and ions hidden for clarity; see Methods). (**C**) Docking of the exogenous oligopeptide agonist deltorphin in the cell surface domain of PDB 8F7S. **Left panel**: 8F7S with docked oligopeptide. **Right Panel**: Docked deltorphin (blue atoms) superimposed on the native cryoEM drug conformer (yellow atoms). The RMSD for the superimposed molecules was 2.8 Å, with the C-terminal Tyr residues showing the greatest misalignment. Docking was carried out using AutoDock VINA with fixed backbone atoms but flexible residue side chains. Despite its complex torsional landscape, AutoDock VINA predicted a reasonable deltorphin conformation. (**D**) Trajectory analysis of the MD simulation for the ACC519TT-GPCR complex (blue line: total system energy (kcal/mol); green line: RMSD of ligand conformation; orange line: receptor RMSD; gray line: ligand movement RMSD). The images under the trajectory graph show the GPCR-bound drug orientation in selected frame captures from the MD simulation. (**E**) Binding mechanism of bisartan ACC519TT extracted from the minimum-energy frame capture of MD simulation. **Left Panel**: 3D ligand-receptor interactions computed by YASARA Structure showing the preferred interaction of the anionic tetrazole groups with positively charged Lys^214^ and Arg^192^ residues. **Right Panel**: A similar drug-binding motif was obtained from 2D interaction plots (Center for Bioinformatics|German Network for Bioinformatics Infrastructure: https://proteins.plus/) (accessed on 28 February 2025). Color key for interactions: green = hydrophobic; blue = cation-pi; red = pi-pi; black dashed line = hydrogen bonds.

The µOR (Figure 7) and δOR (Figure 8) also demonstrate CAD affinities, which are generally higher for ARBs than for opioid ligands, although in this case, the overall ligand affinities (uM) are several orders of magnitude lower than those for AT_1_R or alpha receptors (nM). The significance of this finding is unclear, although it is known that opioid peptides (enkephalins and dynorphins) have affinities in the nanomolar range. Taken at face value, the relatively similar affinities of all opioid ligands in Figure 8 imply that differences in potency (for example, carfentanyl is 1000 times more potent than morphine) are due to differences in efficacy.

Virtual ligand screening was performed targeting the upper cell surface-binding domain of the human GPCR δOR (PDB 8F7S). This particular OR was used for docking since it was recently characterized in terms of its structure and exogenous agonist peptide (deltorphin) drug-binding behavior [40]. Having the deltorphin-activated agonist structure enabled us to compare sartan-induced deviations from the native cryoEM structure after equilibrium MD simulations, offering insights into the agonist/antagonist properties of our experimental sartans (e.g., ACC519TT) (Figure 8).

The drug cohort used for docking comprised 44 ligands spanning a range of exogenous small-molecule opioid drugs (e.g., codeine, oxycodone, naltrexone, quetiapine, and prazosin), several FDA-approved sartans (e.g. candesartan, telmisartan, and irbesartan), and experimental anti-hypertensive sartan-like drugs (e.g., bisartans, including ACC519TT, BisA, and BisB). To account for the binding-energy bias introduced by differences in the size of drugs, binding efficiencies were also computed and expressed as kcal/mol-atom. The docking results shown in Figure 8 indicate a clear trend of better binding for the experimental bisartans and other sartans compared to the small-molecule opioids. While binding efficiencies were lower for larger sartans and bisartans (including ACC519TT), they were generally close to the 0.35 kcal/mol-atom threshold, which is typically considered sufficient to predict a stable drug-receptor complex.

To test the ability of AutoDock to correctly predict the pose of a complex ligand, such as the exogenous peptide agonist deltorphin, this ligand was re-docked into the upper cell surface-binding domain of PDB 8F7S. A poor conformational pose was obtained (RMSD = 8.0293 Å over 110 matched atoms) when deltorphin was docked in a fully flexible mode, i.e., with the peptide backbone and residue sidechains remaining flexible. This result was not unexpected because most commercial and open-source docking algorithms are not optimized to handle the large dihedral space of oligopeptides. However, the docking fidelity of AutoDock was greatly improved by fixing the deltorphin backbone while keeping all residue sidechain atoms fully flexible during docking. When this was done, an RMSD of 3.17 Å was obtained, suggesting that AutoDock can provide a reasonable molecular pose even for complex peptide ligands.

### 3.2. MD Analysis of ACC519TT-Receptor Complex Stability and Binding Motifs

Equilibrium MD simulations were employed to evaluate whether the docked anionic bisartan ACC519TT remained stably bound in three of the GPCRs investigated, including δOR (PDB: 8F7S), µOR (PDB: 6KUW), and AT_1_R (PDB: 6OS0). ACC519TT was chosen as a “model” ligand in MD simulations since it generally scored well in the docking studies across all of the GPCRs examined. Additionally, it is a relatively large and bulky ligand, properties that tend to reduce the per-atom docking efficiency of a drug (i.e., total docking energy divided by the heavy-atom count), thereby yielding a more conservative estimate of complex stability compared to smaller opioid drugs, such as fentanyl or oxycodon.

**Figure 9:**
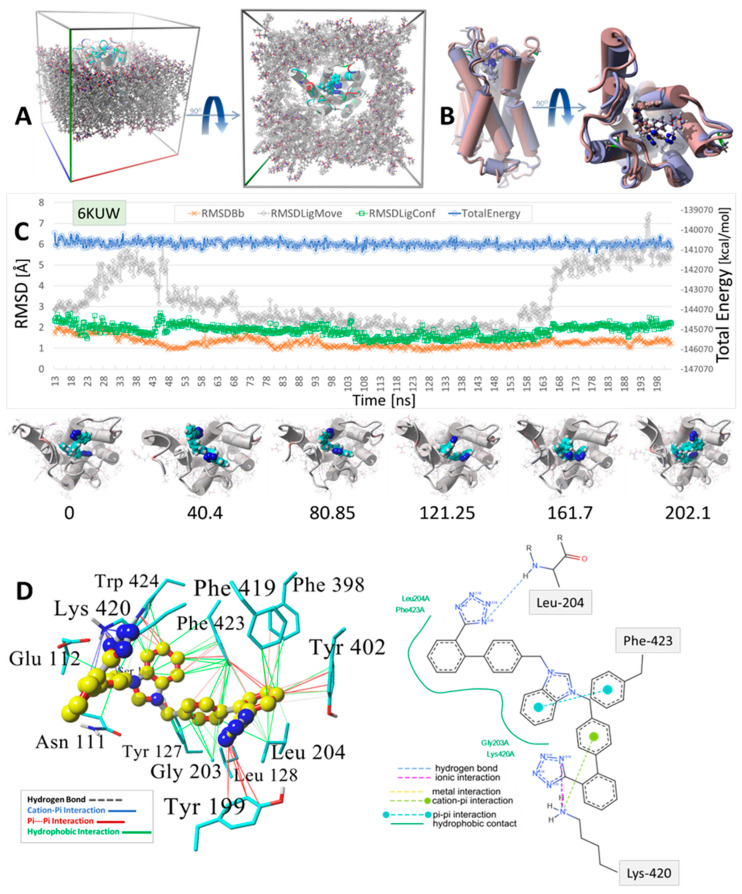
MD simulation was used to determine the stability of the docked drug MD simulation of bisartan ACC519TT docked into the cell surface domain of the human α2C-adrenergic GPCR receptor (PDB 6KUW). (**A**) Setup for the MD simulation showing the periodic cell in oblique (**Left Panel**) and Y-axis (**Right Panel**) projections. The GPCR was embedded into a phosphatidyl−ethanolamine lipid bilayer membrane matrix, and the system was fully solvated using TIP3P water plus added Na^+^ and Cl^−^ ions for cell neutralization (Methods). Water and ions are omitted for clarity. (**B**) Minimum-energy drug-receptor complex frame capture (blue atom coloring) superimposed onto the time-averaged complex structure (maroon coloring) from the MD simulation, indicating significant spatial re-orientation of the energy-minimum drug conformation from the time-averaged conformation. (**C**) MD trajectory analysis for the simulation: blue line = total system energy (kcal/mol); gray line = RMSD of ligand translocation in the binding domain; green line = drug conformation RMSD; orange line = receptor backbone RMSD. Images below the trajectory graph are interval frame captures illustrating ligand movement and conformational rearrangements over the course of the MD simulation. The numbers beneath each image indicate the ns of elapsed simulation time. (**D**) Binding motif of bisartan ACC519TT in the minimum-energy drug-GPCR complex. **Left Panel**: Color-coded intermolecular 3D interactions calculated by the YASARA Structure suite (www.yasara.org; accessed 10 December 2024): green lines = hydrophobic interactions; red lines = pi-pi; blue lines = cation-pi; dashed lines = hydrogen bonds. **Right Panel**: 2D interaction diagram computed from online ligand pose analytical tools (Center for Bioinformatics|German Network for Bioinformatics Infrastructure: https://proteins.plus/; accessed on 28 February 2025) The interactions are color-coded for each image. Both methods captured similar interactions, including the ionic (or cation-pi) bonding interactions of one of the anionic tetrazole groups with Lys^420^. However, the methods differed with respect to the other tetrazole group, with the 2D interaction diagram indicating a hydrogen bond with Leu^204^. This hydrogen bond was not detected in the YASARA computation.

**Figure 10:**
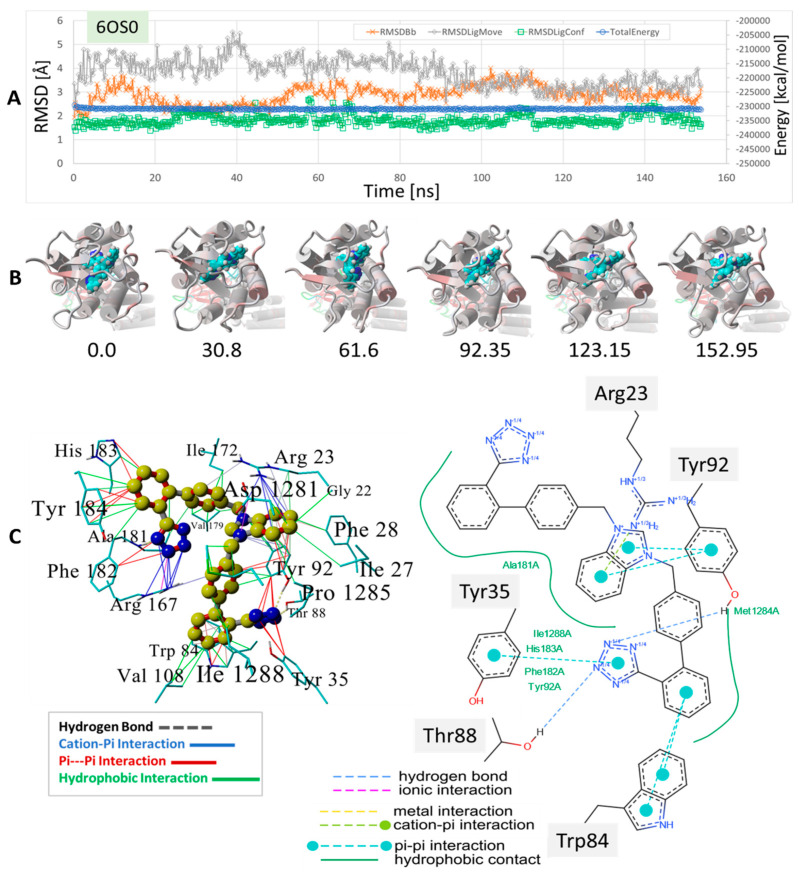
MD simulation to determine the stability of bisartan drug docked to human angiotensin II type-1 receptor (AT_1_R). The setup was similar to that shown for the δOR described in Figure 8 and Figure 9 (see Section 2). The experimental benzimidazole-bi-phenyl-tetrazole, ACC519TT, was docked into the cell surface domain of the membrane-embedded AT1 receptor (PDB 6OS0), followed by an equilibrium MD simulation for 156 ns (see Methods). (**A**) Trajectory analysis showing total system energy (blue line), RMSD of ligand movement (translation) from starting location (gray line), RMSD of ligand conformation compared to starting conformation, and the RMSD of the receptor backbone, all as a function of MD simulation time in ns. (**B**) Frame captures depicting the ligand ACC519TT inside the AT_1_R binding domain near the cell surface. The numbers indicate the ns of elapsed time. (**C**) Mechanistic details of the ACC519TT binding motif in the AT_1_R pocket. Intermolecular interactions were obtained from the minimum energy conformation obtained from MD simulations. Note that the interactions are color-coded differently for the 3D rendition (**left panel**) and the 2D interaction diagram on the right. The benzimidazole group (6-carbon ring fragment) underwent cation-pi bonding with the cationic AT_1_R residue Arg^23^ and hydrophobic bonding with Tyr^92^. One tetrazole group entered into cation-pi bonding with Arg-167, while the other tetrazole group interacted via pi-pi bonding with Tyr^35^ and Tyr^92^. See the text for additional details.

**Figure 11:**
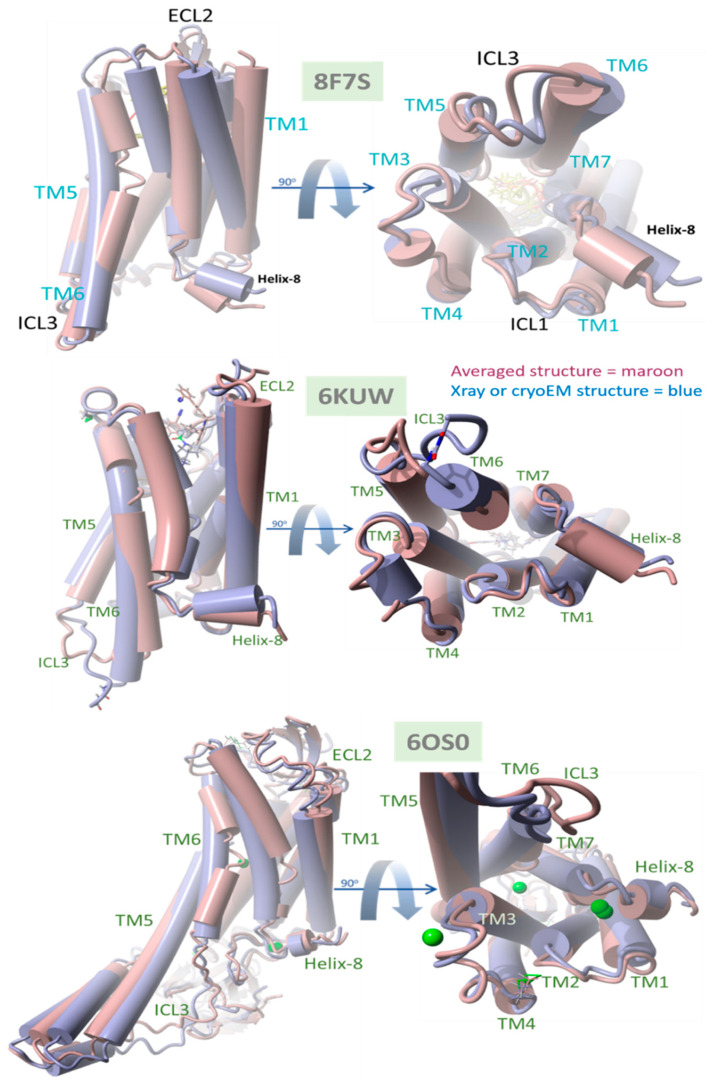
Comparison of native (X-ray crystallographic or cryoEM) PDB structures with time-averaged structures obtained from MD simulations for δOR 8F7S, µOR 6KUW, and AT_1_R, 6OS0. All GPCRs were reported in a ligand-bound agonist configuration (see the text for details). In each case, the experimental bisartan ACC519TT was docked into the GPCR surface domain, followed by MD simulation of the membrane-embedded complex (see Section 2). RMSD values were calculated for the superimposed structures for each system: deltaOR (PDB 8F7S) = 3.08 Å, muOR (PDB 6KUW) = 2.47 Å, and AT_1_R 60S0 = 3.02 Å. The principal ACC519TT-induced distortions observed for 8F7S involved counterclockwise (viewed from above) twisting or rotation of the transmembrane bundle, consistent with driving opioid apo-GPCR structural changes away from the known agonist conformation.

MD simulations were run using an NPT ensemble at 310 °K, pH 7.4, and 0.9 wt% NaCl with additional Na or Cl ions provided for cell neutralization (see Section 2). The GPCRs harboring docked ACC519TT (in the upper cell surface-binding domains) were embedded in a lipid bilayer membrane composed of phosphatidyl ethanolamine. Following the initial system annealing equilibrations, the MD simulations were run for 150 to 200 ns. The results of the MD simulations are presented in Figure 9, Figure 10 and Figure 11 for the δOR, µOR, and AT_1_R. In each case, bisartan ACC519TT remained quasi-stable over the 150–200-ns MD trajectories. Although modest translational motion (RMSDLigMove) and internal conformational changes (RMSDLigConf) of bisartan were observed, the drug tended to remain stably confined within the binding domain.

We also compared the types of intermolecular interactions of ACC519TT for each OR system using the minimum-energy frame capture from each MD trajectory. Due to the enhanced isosteric nature of anionic tetrazoles relative to carboxylate groups (e.g., the former can also adopt efficient aromatic pi-pi bonding in addition to ionic interactions), it was not surprising to observe the interaction of one or both tetrazole functional groups with cationic Arg and Lys residues associated with the binding pocket. This was the case for ACC519TT bound to the δOR pocket of PDB 8F7S, in which one tetrazole exhibited both cation-pi and pi-pi interactions with Lys-214, and the other tetrazole entered into cation-pi interactions with Arg-192 (Figure 8). It is also noteworthy that the positively charged central benzimidazole heterocycle linking the symmetric biphenyl tetrazole groups tended to interact with nearby aromatic δOR residues (e.g., Phe-280) through pi-pi, cation-pi and hydrophobic interactions.

A similar situation was observed for the ACC519TT interaction with the µOR (PDB 6KUW), in which one tetrazole moiety underwent cation-pi ionic bonding with Lys-420. In this OR system, the second tetrazole group interacts with Tyr-199 primarily by pi-pi intermolecular bonding (Figure 9). Similar to the situation with δOR described above, the central ACC519TT benzimidazole was bonded to Phe-423 of µOR via pi-pi and hydrophobic interactions.

A binding motif more or less comparable to those described above for δOR and µOR was also observed for the ACC519TT interaction with AT_1_R, PDB 6OS0 ( Figure 10). In this case, there were at least two AT_1_R Arg residues located near ACC519TT, including Arg^167^ and Arg^1281^. The former (Arg-167) mainly underwent cation-pi interactions with one tetrazole group, while the other tetrazole interacted with µOR residues Tyr^35^ and Tyr^92^ via pi-pi bonding. Note that the 2D interaction plot depicted in Figure 10 also reveals two hydrogen bonds between the second tetrazole group and the hydroxyl groups of the AT_1_R residues Thr^88^ and Tyr^92^. Interestingly, ACC519TT was further stabilized by cation-pi bonding between the AT_1_R residue Arg^23^ and the central benzimidazole functionality of ACC519TT. This type of non-bonded ionic interaction was mainly associated with the 6-carbon aromatic ring of the benzimidazole moiety, which likely carried a negative charge relative to the 5-member ring. Both benzimidazole rings also underwent pi-pi interactions with Tyr-92. Based on the observed binding motifs described above, bisartan ACC519TT (and its close structural relatives) appears to be well-suited for stable binding to the GPCR pockets examined in this study. This characteristic is primarily due to the large torsional space available to these and similar drugs, which enables a high degree of conformational flexibility in their structures. This is demonstrated by the twisted, spaghetti-like shapes consistently adopted by bis-phenyl tetrazoles across all the opioid receptors examined in this study. Further, the carboxylate biosteric behavior and rich resonance properties of the anionic tetrazole group further enhance its ability to bind in versatile ways.

## 4. Discussion

Herein, we show the CAD-binding affinities of various ARBs, αAR, and µOR ligands to the primary agonist binding pocket on the cell surface domains of AT1R, αAR, and µOR. In general, bisartans (A, B, C, and ACC519TT) and certain sartans (telmisartan and olmesartan) were found to have the highest affinity not only for AT1R but also for αARs and µORs and were preferred to their respective agonist ligands (epinephrine and fentanyl) as well as antagonist ligands (prazosin and naltrexone) (Figure 1, Figure 2, Figure 3, Figure 4, Figure 5, Figure 6, Figure 7 and Figure 8). This counterintuitive finding could reflect differences in affinity between uncoupled crystal structures and fully functional receptors coupled with signaling pathways or could be due to an overestimation by CAD of the contribution of multiple aromatic ring structures in ARBs. The bilateral geometry of bisartans, which are zwitterions, may contribute to their enhanced potency. However, these findings may also reflect the actions of ARBs as high-affinity partial inverse agonists of αARs and µORs. The stability of the selected drugs binding to receptors was confirmed by molecular dynamics experiments (Figure 9, Figure 10 and Figure 11), providing confidence in the CAD data.

### 4.1. Evidence for Agonist/Antagonist Behavior of the Bisartan ACC519TT

The data presented in Figure 11 compare the native (X-ray crystallographic or cryoEM) PDB structures with their time-averaged structures obtained from MD simulations for δOR (PDB 8F7S), µOR (PDB 6KUW), and AT1R (PDB 6OS0). These GPCRs have been previously reported to be in a ligand-bound agonist state or configuration [40]. Thus, the significant structural departure of the ACC519TT-bound GPCR from its agonist state following equilibrium MD simulations should be consistent with bisartan functioning primarily as an OR antagonist. Conversely, a slight deviation from the original (native) agonist state may indicate possible GPCR activation by ACC519TT. For each OR, the experimental bisartan ACC519TT was docked into the GPCR cell-surface domain, followed by MD simulation of the lipid bilayer-embedded complex for 156–200+ ns (see previous section and Methods). Following MD, the time-averaged OR structure was superimposed onto the native OR X-ray (or cryoEM for 8F7S) structure, followed by the calculation of the RMSD value. As shown in Figure 11, the most significant RMSD deviation was observed for the δOR (PDB 8F7S) system (RMSD ≥ 3.08 Å), whereas less structural deviations were observed for the µOR (PDB 6KUW; RMSD = 2.47 Å) and AT1R 6OS0 systems (3.02 Å). The principal ACC519TT-induced distortions observed for 8F7S involved counterclockwise twisting or rotation of the transmembrane bundle (viewed from the top-down perspective parallel to the longitudinal axis of the transmembrane bundle). The twisting was most pronounced and clearly observed in the upper (cell surface) 7TM-bundle helices but was less apparent in the bottom-up projection. The results are consistent with ACC519TT functioning as an antagonist with respect to 8F7S but as a possible agonist or partial agonist for 6KUW and 6OS0. Because the observed time-averaged GPCR conformational changes occurred over relatively short MD simulation timescales (~200 ns), the possibility that such changes were solely due to early relaxation of the ligand-GPCR complexes cannot be ruled out. However, it is also possible that the conformational changes might signal the accumulation of stepwise low-energy minima along an agonist-to-antagonist switching trajectory, similar to a process recently described by Palmai et al. [41] for N-methyl-D-aspartate receptors (NMDARs), heterotetrameric ligand-gated GPCR channels involved in neuronal synaptic communications.

### 4.2. AT_1_R and the 3-State Model

**Figure 12:**
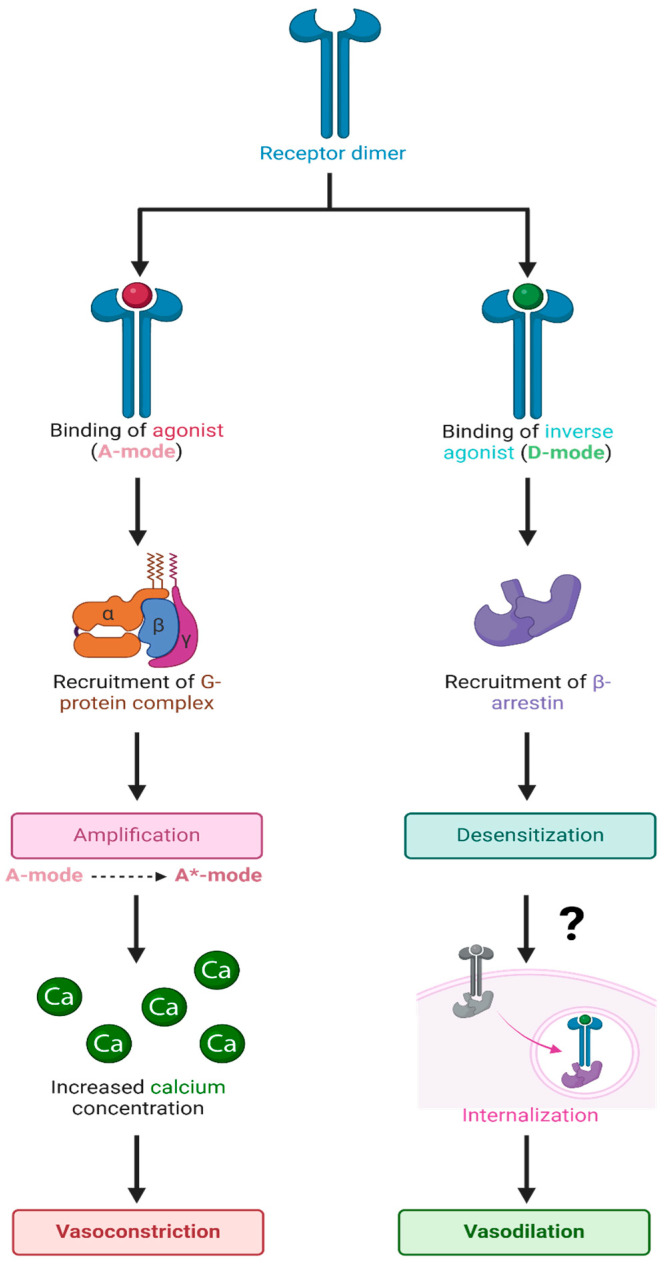
Hypothetical binding of agonists and inverse agonists to GPCR dimers. Schematic representation of the binding of agonists and inverse agonists to overlapping sites on the extracellular binding pocket of the GPCR dimer, resulting in binding in the A-mode and D-mode, respectively, which couple to the corresponding signaling mechanisms for vasoconstriction (amplification) or vasodilation (desensitization), respectively. It is possible that binding in the D-mode involves an allosteric site that is separate from the agonist binding site. For µORs, inositol phosphate replaces calcium ions as a second messenger. Abbreviations: A-mode, resting state; A*-state, activated state; Ca, calcium; D-state, desensitized state. The figure was created using Biorender. L.K.G. (2025) https://app.biorender.com/illustrations/661cf9c7d9e131b4ff8f4bc8.

ARBs are not only inverse agonists at AT_1_Rs but also desensitize these receptors for long periods, most likely by evoking receptor internalization. In addition, ARBs inhibit AT_1_R synthesis [42]. Accordingly, the mechanism of action of ARBs appears to be derived from a different mode of binding (D-mode) to the AT_1_R than that employed by the agonist AngII (A-mode) (Figure 12). The binding of AngII in the A-mode induces an increase in its affinity (from A to A*-mode) by a cooperative mechanism involving receptor dimerization with concomitant G-protein binding, leading to the amplification of the contractile response. The degree of positive cooperativity (Hill coefficient) is a measure of agonist efficacy [43] and is tissue-dependent (e.g., AngII acting at AT_1_R: rat aorta > portal vein > uterus). In contrast, ARBs appear to bind (D-mode) to the same extracellular binding pocket but desensitize the AT_1_R for prolonged periods and couple the receptor to an alternative second messenger (possibly beta-arrestin), which induces relaxation.

The resting state of the AT_1_R and presumably all GPCR has two overlapping binding sites, which engender opposite responses: (1) agonist (positive cooperativity) and (2) inverse agonist (negative cooperativity) accompanied by receptor desensitization. At very high doses, the agonist itself may also bind in “inverse agonist” mode, causing tachyphylaxis and, thus, desensitization; tachyphylaxis, desensitization, and inverse agonism are related phenomena. Desensitization is likely a property commonly associated with inverse agonists for many receptors. In the final analysis, the receptor can exist in three states: the low-affinity resting state (A), the agonist-induced high-affinity activated state (A*), and desensitized state (D).

Structure-activity studies have shown that tyrosinate anion formation in AngII is essential not only for activating AT_1_R but also for desensitizing the receptor. Chemical reactivity studies have shown that the Phe-carboxylate in AngII, despite the availability of stronger interactions with Arg guanidinium, N-terminal amino, or TyrOH, favors interaction with His imidazole, leading to the creation of a tripartite charge relay system (CRS) TyrOH-HisNH—CO_2_ in solution, which is stabilized by interaction with Arg in guanidinium [44]. This tyrosinate conformer consolidates as AngII sheds water molecules upon entering the membrane receptor environment and is consequently available for direct interaction with receptor-based groups, such as R167 and K199 (which interact with an equivalent tetrazole anion in ARBs) [45].

**Figure 13:**
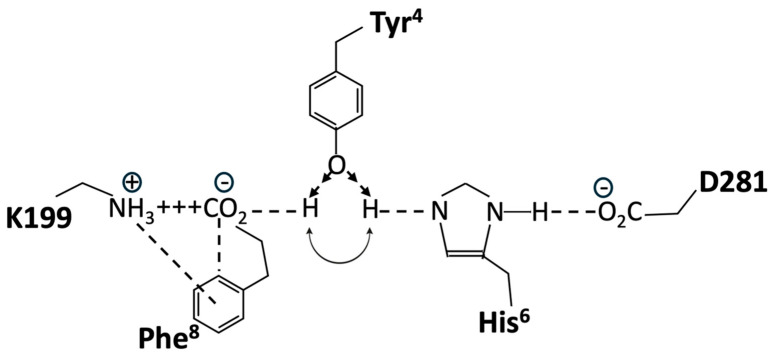
Hypothetical molecular switch for AngII bound to AT_1_R, showing two possible positions for the Tyr^4^ hydroxyl of AngII. Interaction with His^6^ imidazole forming a CRS is predicted to activate the receptor (A-mode) and is favored in the presence of the Phe^8^ ring quadrupole (shown). Interaction with Phe^8^-carboxylate, which forms a salt bridge with K199 (D-mode); the default position in the absence of the Phe^8^ ring (e.g., for sarilesin binding). Single-letter abbreviations for amino acids denote AT_1_R residues.

Crystallography studies [46] show that the binding of AngII to AT_1_R is accompanied by a conformational rearrangement in which the Phe^8^ carboxylate forms a salt bridge with K199, Tyr^4^OH binds to Phe^8^-carboxylate, and the role of Phe^8^-carboxylate in CRS prior to binding is essentially replaced by D281 at the receptor. Thus, realignment of the His^6^ imidazole of AngII upon binding to the receptor results in the interaction of the imidazole with D281 of AT_1_R, which provides the opportunity to recreate the CRS in the receptor-binding pocket because the Tyr4 hydroxyl of AngII can move from interaction [46] with the Phe^8^-carboxylate (which has formed a salt bridge with D199) to interact with the His^6^ imidazole N (Figure 13). Thus, re-establishment of the CRS (Tyr^4^OH--His^6^NH--D281, resulting in tyrosinate formation) could represent binding in the A-mode (which is the trigger for receptor dimer cooperativity (A*-mode)). For the two poles of the switch shown in Figure 13, in both cases, TyrOH interacts with carboxylate, albeit indirectly through the electrical conduit provided by His imidazole at one pole and through the partially neutralized [by K199] Phe-carboxylate at the other pole. Interestingly, the CRS conformation of AngII documented in solution is reenacted at the receptor and may provide clues/insights into other peptide-receptor interactions.

A wealth of structure-activity data is available for AngII, all of which must be accounted for by any proposed receptor mechanism. Accordingly, methylation of the Tyr^4^ hydroxyl of angiotensin analogs would prevent interaction with either pole of this hypothetical molecular switch, resulting in the observed deactivation of both agonist and inverse agonist analogs (notably, sarmesin is a competitive antagonist because it binds but does not activate AT_1_R, and methylation of the Tyr^4^ hydroxyl of the inverse agonist sarilesin is likewise inactivated). The role of Phe^8^ is also a case in point because its replacement with an aliphatic sidechain (Ala, Ile) produces an inverse agonist. The quadrupole moment of the Phe^8^ ring (which has negative charges above and below the ring and a positive charge in the plane of the ring) permits the simultaneous interaction of the ring with the positive charge of K199 and the negative charge of Phe^8^-carboxylate in the salt bridge (Figure 13). This interaction with Phe^8^-carboxylate is associated with agonism (A-mode) and may be important for promoting one position of the gating mechanism, namely, repositioning TyrOH to form the CRS conformation at the receptor (A-mode). In contrast, analogs like sarilesin, which have an aliphatic sidechain, may favor the interaction of TyrOH with the Phe-carboxylate in the K199 salt bridge, the inference here being that the quadrupolar interaction of the Phe^8^ ring with the salt bridge is enough to bias the gating toward the CRS conformation (A-mode). Thus, the bonding of TyrOH to Phe^8^-carboxylate is not energetically favored when the ring quadrupole is present. In addition, the crystal structure of saralasin [46] shows that the Arg^4^ guanidinium group interacts with D281, probably weakening the interaction of D281 with His^4^ of AngII and compromising CRS formation (thereby favoring the D-mode).

The crystal structure of AngII bound to AT_1_R shows the interaction of Tyr4 OH with Phe^8^ carboxylate and has not revealed a CRS interaction involving Tyr^4^ OH with His^6^ imidazole, possibly because the formation of the CRS requires the presence of G protein and/or other cofactors. In this regard, AT_1_Rs exist in dimer form within the cell membrane, allowing agonists to invoke homotropic cooperativity, which is intimately associated with heterotropic cooperativity due to interaction with the G protein, such that the agonist can induce an increase in its own affinity, thereby amplifying the response over a narrow concentration range. The absence of the G protein would obfuscate this trick and possibly prevent the binding of the agonist in the first place. Molecular dynamics simulations of biased signaling mechanisms [47] have not revealed CRS interactions at the receptor, possibly because ligand-dependent conformational changes occur on time scales longer than those of the simulations.

The dose-response curve for AngII is bell-shaped [21], implying that at a certain concentration or response level, AngII switches from an agonist to an inverse agonist. It is difficult to explain how both agonist and inverse agonist activities are manifested by the same peptide molecule interacting at the same receptor-binding site. Possibly yet unidentified intracellular signaling entities control this form of biased agonism by directing the receptor conformation responsible for agonism (contraction, A-mode) toward another conformer responsible for inverse agonism (relaxation, D-mode) when agonist/response levels reach certain limits. Whereas angiotensin peptide agonists and inverse agonists interact principally with K199 of AT_1_R [46], nonpeptide ARB sartans appear to interact with R167 (Figure 2).

### 4.3. Biased Agonism—Agonist Versus Inverse Agonist Binding

GPCRs can be viewed (Figure 12) as having two overlapping binding sites and can bind ligands in two possible ways: A-mode is for agonists, and D-mode is for inverse agonists, the latter desensitizing/inactivating the receptor for prolonged periods, most likely by dissociation of the ligand from arrestin and signaling internalization step (Figure 12). Competitive antagonists bind only in the A-mode and essentially reduce the effective concentration of the agonist. Tachyphylaxis occurs when the agonist reaches a sufficiently high concentration to start binding in D-mode and perhaps exhausts the supply of certain intracellular components (e.g., G protein) and/or induces internalization. The mechanism parallels that of enzyme action, where the agonist substitutes for the “substrate”, the response is equivalent to the “product”, and an inverse agonist is a “noncompetitive inhibitor”, but there is no covalent bond cleavage.

In the absence of receptor dimers and G-protein interactions, as is the case for the crystal structures investigated herein, all agonist ligands shown in Figure 1, Figure 2, Figure 3, Figure 4, Figure 5, Figure 6 and Figure 7 (i.e., AngII, epinephrine, and enkephalin/dynorphin) are presumed to bind to the resting inactivated low-affinity state of the receptor (A-mode). This is likely the low-affinity site observed in binding studies in the presence of guanosine triphosphate (GTP), as GTP uncouples the receptor from signaling and prevents cooperative activation. The ‘GTP-shift’ is the difference in agonist affinity between the activated (nanomolar) and resting (micromolar) states of the receptor and reflects agonist efficacy. The cooperative activation phase of an agonist manifests as the “threshold” effect seen in pharmacological assays, and above this threshold, the dose-response relationship follows first-order kinetics until tachyphylaxis occurs. Any candidate ligand that is not an agonist and, therefore, does not have the specialized ability to induce dynamic cooperative agonist action (i.e., A-mode to A*-mode) still has the possibility of binding to the active site of a receptor in desensitization mode (D-mode) (Figure 12). Most of the high-affinity ligands shown in Figure 1, Figure 2, Figure 3, Figure 4, Figure 5, Figure 6 and Figure 7, which are not known agonists for each receptor, probably bind in the D-mode; however, some competitive antagonism in the A-mode cannot be ruled out. The data in Figure 1, Figure 2, Figure 3, Figure 4, Figure 5, Figure 6 and Figure 7 suggest that binding in the D-mode can be indiscriminate and that certain ligands unrelated to the natural agonist can demonstrate substantial affinity for the D-site of several GPCRs. It is possible that certain ARBs, particularly zwitterion bisartans that have higher affinities at all three receptors investigated, are overestimated by the CAD software; however, it is known that ARBs can modulate the contractile response to phenylephrine in isolated vascular smooth muscle [21]. Conversely, it is possible that an αAR inverse agonist, such as prazosin, could modulate the response to AngII in the same tissue, given the permissive nature of the crossover binding elaborated herein. Reciprocal modulation could be relevant in an environment (e.g., hypertension) where the same physiological outcome from both receptors (i.e., lowering blood pressure) is desirable. This may explain why critical hypotension can develop in conscious rabbits administered an exceedingly low dose (1 pg intravenous administration via the auricle artery) of telmisartan [21]. Therefore, if telmisartan acts on αARs in addition to AT1Rs, a potential compensatory mechanism is also desensitized. However, the µORs, which presumably have a different endpoint, and the possible role of ARBs in moderating receptor function are less obvious. Candesartan and valsartan have been shown to inhibit morphine tolerance in microglial cells [48] and rats [49].

### 4.4. Alternative ARB Binding Site

It is not just membrane receptors that are prone to binding and responding to ARBs, as CAD simulations have predicted ARB binding to proteolytic enzymes such as neprilysin [50] and angiotensin-converting enzyme 2 [10]. ARBs can also inhibit SARS-CoV-2 infection [10], and there is evidence that other respiratory viruses (i.e., influenza and RSV) are prone to inhibition by ARB actions at non-structural protein 3, inhibiting ADP-ribose binding [11]. ARB binding sites are ubiquitous, raising the possibility that a natural ligand may exist that targets these sites. Indeed, it has been proposed that the inverse agonist analog, EGVYVHPV, encoded by the (−)mRNA strand, which is complementary to the (+)RNA strand encoding AngII, could be a natural ligand for the ARB binding site [51].

### 4.5. Structural Considerations for Ligands Binding to GPCR

Compounds that show an enhanced ability to cross-bind to all three GPCRs include ARB sartans (e.g., telmisartan) and bisartans (e.g., ACC519TT), alpha-blockers/inverse agonists (e.g., prazosin), and µOR ligands (e.g., fentanyl) (Figure 1, Figure 2, Figure 3, Figure 4, Figure 5, Figure 6 and Figure 7). These compounds all share common structural properties, namely the presence of a positively charged cyclic amino/imino group coupled with a lengthy hydrophobic region. Sartans contain imidazole, bisartans contain imidazolium, prazosin contains piperazine, and fentanyl contains piperidine. Bisartans tend to demonstrate greater binding affinities than sartans at all GPCRs (Figure 1, Figure 2, Figure 3, Figure 4, Figure 5, Figure 6 and Figure 7), which may be due to the presence of a formal positive charge (imidazolium), whereas sartans contain only a partial charge (imidazole). In contrast, the tetrazole or carboxyl group(s) present in all sartans/bisartans seem to be a less significant contributor to binding to the αAR (Figure 3, Figure 4, Figure 5 and Figure 6) and in particular, the µOR (for ACC519TT, both tetrazoles point up and away from contact with the surface of the µOR binding pocket, as shown in Figure 4). However, for both αAR and µOR, the hydrophobic nature of the binding pockets does not appear to be particularly compatible with charged groups. Previous studies have suggested that opiate ligands (and possibly the N-terminal of enkephalin peptides) may bind deeper into the transmembrane helices of the µOR and include a salt bridge to D147 [36]. However, since our studies involve the uncoupled resting state of the receptor, it is possible that the interaction with D147 represents the agonist activation step (A*-mode), providing the high-affinity form of the actionable dimer and G-protein. Transitioning the receptor from a resting low-affinity A-state to a high-affinity A* state requires energy, which can be supplied by salt bridge formation. Alternatively, the AT_1_R (Figure 2) provides a binding pocket containing ionic sidechains, which could accommodate both cationic and anionic groups present in AngII and ARBs; however, in these CAD studies, salt bridges do not seem to be directly formed. However, when molecular dynamics simulations are subsequently applied, the ion pair consolidates. As outlined above, reaching for interaction with R167 of the receptor may be associated with the agonist-induced cooperative activation step, resulting in a high-affinity receptor dimer and G-protein (binding in A*-mode).

### 4.6. Permissive Crossover Binding at GPCR

Evolutionary considerations could apply to the GPCR family of receptors, which likely evolved by divergence from a single ancestral receptor. The present findings suggest that several GPCRs can bind ARB ligands with unusually high affinity and that the primary binding site, which coincides/overlaps with the agonist binding site for each GPCR, may be associated with inverse agonism and receptor desensitization/tachyphylaxis (Figure 12). Since there is no obvious sequence homology among the cell surface active sites of the three GPCR investigated and the binding of ARB at each receptor involves different amino acid contacts in each case (Figure 1, Figure 2, Figure 3, Figure 4, Figure 5, Figure 6 and Figure 7), it is possible that functionality has been maintained for all three GPCR more by the general architecture of the electrostatic surface features than by structural integrity. However, investigations of 3D electrostatic surface maps of the primary binding sites of the three receptors have revealed limited general similarities in this regard. Different GPCRs assign the same functional rules to different residues (i.e., functional switches). Nevertheless, it represents an extraordinary feat to evolve an active site that is highly selective for one natural ligand while apparently maintaining the ability to bind other common entities across a family of GPCR. If membrane receptors act as cellular gatekeepers, they appear to exhibit flexible recognition criteria for binding or participate in highly intricate cross-binding regulatory mechanisms. One potential explanation is that molecular modeling and docking simulations conducted using computer software may introduce artificial biases into these calculations. Additionally, it is plausible that desensitization involves ligand binding to multiple sites on the same receptor molecule. Comprehensive mapping of αARs identified two low-affinity allosteric sites in addition to the agonist binding pocket (Figure 3). Furthermore, another site situated in the cleft between symmetrical receptor dimers (Figure 5) appears capable of binding ARBs and alpha-blockers but not alpha agonists, potentially representing the D-site described herein.

The present findings suggest a profound lack of specificity for GPCR binding of small-molecule medicines compared to their peptide counterparts. Investigations of angiotensin peptide analog binding to AT_1_Rs have demonstrated that extreme structural integrity is required for both agonist activity and receptor desensitization [50]. For example, the competitive peptide antagonist sarmesin can block AngII-induced contraction (A-mode), but not sarilesin (peptide inverse agonist) induced desensitization (D-mode). Similar high-specificity structure-activity considerations apply to enkephalins/dynorphins binding to opioid receptors. This high selectivity for peptide binding is likely a feature of their flexibility and hydrophilicity, which enables conformational sampling on an epic scale, along with certain fragilities (e.g., membrane incompatibility and enzyme susceptibility) that accompany this property. Moreover, tyrosinate fluorescence lifetime studies have shown that there is a precedent for considering the influence of membrane dielectric properties in promoting the bioactive conformations of peptides [45] when they are present at membrane receptors. In this regard, Ang II is not the only peptide known to create tyrosinate anions for receptor interaction, as a family of “tyrosinate hormones” are known to exist, including vasopressin, oxytocin, gonadotropin-releasing hormone, and enkephalin [52]. The relatively rigid hydrophobic structures of small-molecule mimetics allow them to penetrate the surface of membrane proteins and bind in a permissive manner. It may be relevant that opiate antagonists, like naltrexone, can moderate addiction to substances other than opiates (e.g., alcohol) and perhaps reveal naltrexone as an inverse agonist that can exhibit crossover binding with alternative receptors. Naltrexone is a glial cell modulator that exhibits anti-inflammatory activity [53]. Notably, ARBs inhibit morphine tolerance and inflammation in microglial cells [48] and morphine tolerance in rats [49], suggesting the cross-binding of ARBs with opioid receptors. It is significant that ligands that cross-bind to different receptors are often blockers (D-mode) rather than agonists (A-mode), reinforcing the premise that the process of receptor stimulation can be much more sophisticated and discriminating than receptor blockade. Hundreds of ARB analogs have been synthesized, none of which are agonists. These predictions from CAD indicate that the strongest possible non-covalent interactions available, namely salt bridge formation between acids and bases, are not present for the binding of ligands to the receptors, as shown in Figure 1, Figure 2, Figure 3, Figure 4, Figure 5, Figure 6 and Figure 7. However, as outlined above, such interactions may occur during receptor stimulation or the A*-mode. Instead, all binding energies obtained from these binary interactions are contributed by a host of relatively weak interactions. This could reflect the hydrophobic nature of the binding pockets of αARs (Figure 3, Figure 4, Figure 5 and Figure 6) and µORs (Figure 7).

The concept of “induced fit” may apply mainly to peptide ligands and have less value for the binding of nonpeptide mimetics. The structures of ARBs are more rigid and dependent on the architectural geometry of the binding site, in stark contrast to the dynamics of peptide interactions. In fact, there is so little in common between peptide and non-peptide ligands that it is possible that the clinical effects of ARBs are mediated by a different site altogether, which could be on the receptor, on a component of the signaling mechanism or even at the level of biosynthesis of new proteins/receptors. For example, candesartan inhibits AT_1_R expression in microglial cells [48].

### 4.7. Bioassays Versus CAD

It is notable that certain ARB sartans/bisartans can completely block the smooth muscle response to AngII; however, these drugs cannot completely block phenylephrine-induced contraction [21]. For example, in isolated rabbit iliac arteries, candesartan (10^−6^ M) completely obliterates the response to AngII but achieves only a partial block (20–30%) of the phenylephrine response [21]. However, extremely low concentrations of ARBs (10^−40^ M) also partially block α1ARs, which is not consistent with actions at the A-site because competitive inhibition should be concentration-dependent and saturable but could be mediated by actions at the D-site (Figure 12). αAR inverse agonists, such as prazosin, can completely block the alpha agonist bioassay response, indicating that they are in one sense more potent than ARBs at αARs. Conversely, ARBs like candesartan, which partially block the phenylephrine response at doses as low as 10^−40^ M, suggest that ARBs may have to be defined as super high-affinity partial blockers, or partial inverse agonists.

CAD studies of αARs predict that ARBs, like candesartan, have higher affinities than αAR blockers/inverse agonists, like prazosin and doxazosin (Figure 3, Figure 4, Figure 5 and Figure 6), which contradicts the apparently high affinities (super-low doses) of ARBs in rabbit isolated artery α1AR organ bath studies [21]. However, mass action laws probably do not apply to irreversible (desensitizing) ligands, and affinity constants derived from bioassay data probably have no real meaning. This is because the binding affinity is conventionally represented by the equilibrium dissociation constant, which is the ratio of the rate of dissociation to the rate of association. Desensitization, by definition, is an essentially irreversible process and probably results from the internalization of receptors due to the binding of the inverse agonist sarilesin, which causes AT_1_R to “disappear” from rat uterine intact tissue but not from isolated membranes. When receptors are internalized, the “off” rate becomes infinitesimally small, and therefore, so does K^D^, and the binding affinity consequently appears to be greater. As a result of this, the effect of inverse agonists is time-cumulative and can manifest at extremely low doses [21]. It is likely that desensitization/internalization is a property associated with many inverse agonists at numerous receptors. Likewise, sartans/bisartans appear to have consistently higher CAD affinities at opioid receptors than traditional narcotic agonists like fentanyl and opioid antagonists like naltrexone (Figure 7); however, it seems unlikely that ARBs will be more complete blockers than naltrexone in opioid bioassays. However, ARBs may also be high-affinity partial inverse agonist blockers at opioid receptors (analogous to alpha receptors). In this regard, valsartan and candesartan have been shown to inhibit morphine tolerance in rats [49] and microglial cells [48]. Overall, these CAD findings, which show that ARBs have higher affinities for alpha and opioid receptors, respectively than known potent ligands (Figure 3, Figure 4, Figure 5, Figure 6 and Figure 7), although counterintuitive, appear to agree with bioassay data demonstrating that ARBs are partial inverse agonists that give the appearance of having unusually high affinities for αARs and µORs. Due to the absence of cellular signaling mechanisms, CAD-derived affinities reflect the binding of agonists (A-mode) and inverse agonists (D-mode) to the resting state of the receptor. The transition of the receptor from the resting low-affinity state (A) to the high-affinity state (A*), which is driven by agonist-induced cooperative receptor dimer interactions, requires energy and may be derived from the formation of stronger interactions (salt bridge) with the receptor, which could, in turn, change the interaction with GPCR ligands in bioassays.

Fundamentally, what you see with CAD are “dead” receptors and what you see in bioassays are “live” receptors, and comparisons are difficult. If it were possible to simulate the presence of cellular signaling components, some of the CAD parameters would likely change, but most importantly, the ability to simulate the presence of the agonist. This would create a more dynamic situation, including a third excited state of the receptor (A*), accessible to all ligands. For example, the presence of an agonist might bring R167 of AT_1_R and D147 of µOR into the frame of accessibility for ARBs, and other inverse agonists are not normally able to interact with these amino acid sidechains in the resting state of the receptor. No doubt the CAD values for many of these ligands would change substantially as the situation approaches bioassay conditions, and it would become possible to explain some of the discrepancies between CAD values and bioassays, and in particular, some of the extreme bioassay potencies of ARBs observed when interacting with AT_1_Rs and αARs.

### 4.8. Applications and Limitations of CAD

The perspicacity and value of CAD simulations are demonstrated by the ability to reveal the favored binding site for ligands at the expected extracellular binding domains for all three GPCRs investigated (Figure 1, Figure 2, Figure 3, Figure 4, Figure 5, Figure 6 and Figure 7). Each binding pocket on each GPCR also binds ligands crossing over from the two alternative GPCRs, albeit with lower affinity. It is anticipated that other lesser binding sites revealed by CAD, such as the transdimer site of the αAR (Figure 6), may eventually be assigned functions. There may also be value in the rank order of ligand binding associated with each GPCR, although a more detailed investigation using bioassays is required to support this. To date, bioassays conducted using rabbit iliac arteries with several ARB sartans (i.e., candesartan and telmisartan) and bisartans (i.e., bisartan B and ACC519TT) have not revealed any major discrepancies [21]. However, it is difficult to assign meaningful binding affinities from bioassay data that involve desensitization. Other CAD predictions regarding the cross-binding of αARs to AT_1_Rs and opioid receptors, as well as the cross-binding of opiates to AT_1_Rs and αARs, have yet to be proven in bioassays.

Proprietary software applies semi-empirical energy calculations (such as enthalpy or heat of formation) and, where applicable, ab initio energy calculations to compute the free energies associated with ligand binding. It utilizes progressive search algorithms to identify low-energy conformers. The boundaries are defined by the choice of parameter subsets. Individual electrostatic interactions can be diluted by drug settling into an energy well, where such interactions are minimized compared to the collective influence of other competing interactions. The binding energies derived are used to calculate the ligand affinity using the Arrhenius equation but obfuscate the entropy term implicit in the Gibbs free energy equation. The restrictive geometry of the binding pocket may play a role in these outcomes, such that entropy considerations could take on increased significance. At present, CAD studies, which are only as good as the underlying software programming, can provide useful qualitative insights and general trends into molecular interactions at GPCRs but are yet to be proven quantitatively accurate. Furthermore, just because these ligands appear to bind does not necessarily mean they produce a response, as in theory, ARB binding to the preferred extracellular agonist site could be mute, while lower affinity binding at the transdimer site (Figure 5) may evoke a desensitization response. Moreover, a partial response may cause consequential effects (i.e., ARBs can be inverse agonists at angiotensin receptors but act as partial inverse agonists at αARs and opioid receptors).

ARBs are specifically designed for AT1R, αAR, and µOR, which could be partly due to their CAD-assigned properties; the best binders tend to contain the most aromatic rings (e.g., bisartans > sartans), and notably, the ARB bisartan ACC519TT binds exceptionally well to all three GPCR. Aromatic ring currents create a quadrupole moment with negative charges above and below the plane of the ring and a positive charge in the ring plane, which causes pairs of aromatic rings to form either a slipped parallel plate interaction (as in the DNA helix) or a perpendicular plate interaction [54]. This quadrupolar property allows the electrostatic engagement of aromatic rings with other charged groups rather than participation in hydrophobic interactions. However, ring:ring and ring:dipole interactions are not obviously present in Figure 1, Figure 2, Figure 3, Figure 4, Figure 5, Figure 6 and Figure 7; instead, there is a preponderance of assigned hydrophobic bonds between ligand and receptor-based groups (e.g., as shown individually in Figure 2). The CAD software might treat the biphenyl groups in these compounds primarily as large hydrophobic entities rather than purely electrostatic components despite considering the angle of offset between the biphenyl groups, which suggests some electrostatic considerations. The shape and adhesive properties of the aromatic rings of ARBs evidently play a significant role in their binding to GPCRs.

### 4.9. Coupling of Receptors to Signaling Molecules

It is feasible that the observed binding affinity of ARBs at the D-site (calculated by CAD) could change when the ligand-receptor complex enters the cell (i.e., the observed affinity at the D-site may differ substantially from the actual effector affinity measured by bioassays). Accordingly, virtual uncoupled crystal structures would display different kinetic behaviors from fully functioning coupled receptors. There can be little doubt that the state of coupling of receptors to second messengers is likely to affect the binding characteristics of ligands. Crystal structures have been reported for receptor monomers (Figure 1), receptor dimers (Figure 5), and receptor monomers attached to G-proteins (Figure 7), all of which could influence the binding kinetics of ligands. An extensive array of proteins/receptors is known to interact with *α*AR, adding an unprecedented level of complexity to the number of inputs possible for the presentation of the receptor to the ligand, any or all of which could affect the binding affinity. Accordingly, the data shown in Figure 1, Figure 2, Figure 3, Figure 4, Figure 5, Figure 6 and Figure 7 must be considered with these potentially complicating factors. As it turns out, there is not much difference in the CAD affinities for ligands between monomer and dimer forms of the *α*AR (Figure 4 and Figure 5); however, these crystal structures only partly reflect the reality of the cellular environment since cooperative second messenger interactions are absent. As mentioned above, this may explain the differences between CAD-derived affinities at uncoupled crystal structures versus bioassay data for fully functioning receptors that are coupled to their signaling pathways, e.g., ARB at *α*1ARs: 10^−12^ M for CAD (Figure 4) versus 10^−40^ M for bioassays [21], although an argument could be made that the irreversible nature of desensitization in bioassays probably renders the apparent affinity derived from bioassays invalid because mass action laws are not applicable.

There is a reason to consider that CAD-derived affinities, which are not influenced by irreversible processes such as internalization, might provide more realistic (mass action) values. These could be particularly useful for evaluating desensitizing ligands, along with the resting-state affinities of agonist ligands, which are unaffected by cooperative excitation effects. However, ARB binding and complete desensitization may be significantly enhanced only when the receptor is activated, and a salt0bridge is engaged by agonist binding. Therefore, while the affinities of the desensitizing ligands shown in Figure 1, Figure 2, Figure 3, Figure 4, Figure 5, Figure 6 and Figure 7 are notable, they could be considerably amplified during receptor activation. Considerations like these may help elucidate the exceptional potency of ARBs in bioassays.

### 4.10. Possible Relevance for Addiction

The high-affinity binding of ARBs to alpha and opioid receptors suggests that ARBs may affect the mode of action of natural ligands (i.e., epinephrine and enkephalin) at their respective receptors. There is a known relationship between tolerance/desensitization, dependence, and withdrawal during the addiction process. Suppose the binding of these small molecules is accompanied by receptor desensitization or resensitization, as appears to be the case for ARB binding to α1ARs in smooth muscle tissue studies [21]. There may be an opportunity to investigate the potential application of ARB s/bisartans in the treatment of methamphetamine addiction, where interference in the development of tolerance/desensitization could be exploited [22]. Such considerations also apply to opiate addiction because ARBs, like valsartan and candesartan, are known to inhibit morphine tolerance in rats [49] and microglial cells [48]. Desensitization may be a general mechanism by which inverse agonists, like ARBs, prazosin, and naltrexone, elicit effects at their respective receptors by binding in the D-mode (Figure 12), and this process may be permissive enough to enable cross-binding of ligands among receptors. The question arises as to whether crossover binding to the D-site (Figure 12) can effectively prevent the binding of the formal desensitizing ligand in the D-mode without necessarily interfering with binding in the A-mode. Recent findings support this possibility because, unlike most sartans (i.e., candesartan and telmisartan) and bisartans (i.e., ACC519TT and bisartan B), which tend to reduce the contractile response to phenylephrine in isolated iliac arteries, ACC519T (10^−6^ M) was found to increase the sensitivity of phenylephrine-induced contraction [21]. This suggests that ACC519T may resensitize alpha receptors, presumably by inhibiting/reversing the desensitization process. If this is correct, then it should be possible to reduce tolerance and, thereby, dependence on addictive substances by selectively resensitizing receptors, even with ligands that are structurally unrelated to the natural agonist (such as ACC519 at αARs). Physical and psychological dependence may be mediated by the desensitization of peripheral and central nervous system receptors, respectively; therefore, any resensitizing compound should be able to cross the blood−brain barrier [22].

It is known that opiate ligands, such as naltrexone, can alleviate the symptoms of addiction to substances other than opiates. Thus, there is a precedent for investigating potential crossover binding, particularly for sartans/bisartans, to a variety of receptors associated with addictive chemicals, including opiates. In this regard, ARBs have been shown to attenuate morphine tolerance in rats [49] and microglial cells [48].

### 4.11. Opiate Addiction

Opiates are often associated with a higher level of addiction than most other substances. Interestingly, and perhaps relevantly, it is an anomaly that ligand affinities for the opioid receptor (Figure 7) are several orders of magnitude lower than those for AT_1_R (Figure 1 and Figure 2) or αAR (Figure 3, Figure 4, Figure 5 and Figure 6). There is also insufficient discrimination between the affinities of morphine, fentanyl, and etonitazene to account for their potency differences (1×-100×-1000×, respectively) at the opioid receptors. This could be an example of the vagaries of nonpeptide mimetics acting at receptors intended for peptide ligands that can penetrate membranes, resulting in unconventional kinetic behavior. However, the low affinity/high efficacy nature of opiate agonist interactions sets the opioid receptor apart and may account for its highly addictive properties. It is notable that ligands with very different potencies (morphine, fentanyl, and etonitazene) have similar addiction profiles. Applying the model depicted in Figure 4, low-affinity binding of fentanyl or etonitazene at the A-site apparently signals high agonist efficacy and much higher efficacy than that of morphine. Efficacy is determined only by the degree of cooperativity at the receptor dimer (activation of A to A* resulting in amplification of the response), which is expressed as the Hill coefficient (nH values are log scale between 1 and 2, for example, if morphine nH = 1.5, then for fentanyl nH = 1.7 and for etonitazene nH = 1.8).

Desensitization (equivalent to tolerance, dependence, withdrawal, and addiction) may be due to interaction at the D-site, and the relative affinities of an agonist for the A-site versus D-site could influence addiction properties. Thus, when the affinity for the A-site is much higher than that for the D-site, an agonist concentration sufficient to elicit a response will have little tendency to bind to the D-site and, therefore, little tendency at low doses to elicit desensitization. However, when agonist affinity is similar at the A- and D-sites (A/D ratio ~1), the competing interactions will include desensitization in addition to agonist effects at the receptor. Accordingly, if morphine, fentanyl, and nitozene have similar affinities at A- and D-sites (addiction ratio ~1), they will demonstrate similar addiction properties (despite huge efficacy differences). Thus, the comparatively low affinities of opiate agonists for the opioid receptor relative to other GPCRs may be associated with addiction potential because an unusually low affinity at the A-site brings an alignment with the low affinity of the D-site. Due to these A/D ratio considerations, addictive compounds tend to be partial agonists, so even the exceedingly weak partial agonist morphine retains a high addiction propensity.

ARBs, like candesartan, reduce tolerance to opiates, appear to act as competitive inhibitors at the D-site of the opioid receptor, and probably do not interact substantively with the A-site. Similarly, ARBs appear to interact mainly with the D-site of the αAR, altering the level of desensitization/resensitization but presumably not eliciting addiction. Likewise, ARBs acting on angiotensin receptors only bind to the D-site and have no agonist activity; consequently, they should not be addictive. Indeed, it seems unlikely that the relief felt by hypertensive patients due to the normalization of blood pressure would be considered an addiction. It is likely that desensitization alone does not lead to addiction but is linked to receptor activation. In other words, binding at the D-site must be accompanied by binding at the A-site to elicit the addiction process. This implies that there is a crosstalk between the signaling mechanisms associated with the A- and D-sites, otherwise known as biased agonism.

### 4.12. Pharmacological Context

ARBs demonstrate remarkable potency at angiotensin receptors and *α*ARs in smooth muscle, potentially due to irreversible binding, leading to cumulative effects even at very low doses. These unconventional blocking actions occur at concentrations below the Avogadro limit, as low as 10^−60^ M, and are possibly influenced by quantum effects [21]. Additionally, unspecified perturbations or vibrations in the receptor environment could have contributed to the present findings. It is clear that the physiological effects of ligands can manifest at concentrations far below those normally investigated in isolated tissue pharmacological assays. Classical pharmacological studies are usually conducted above the threshold concentration required to cooperatively activate receptors, and cooperativity may represent a specialized event allowing the organism to ramp up the reactivity of the cell in an urgent situation and, therefore, may not necessarily reflect normal everyday physiological functioning. For example, ACC519T decreased mean arterial pressure in conscious rabbits at femtomolar (10^−15^ M) concentrations [21], creating life-threatening hypotension. Fortunately, rabbits differ from humans because much higher and comparatively massive doses of ARBs do not cause critical hypotension in humans.

### 4.13. The Role of Tetrazole and the Unique Properties of Imidazole and Benzimidazole Based Bisartans/Sartans

Pioneering research on the design and synthesis of losartan analogs led to the discovery of a new class of ARBs in which the imidazole substituents, butyl, and hydroxymethylene groups are at reversed positions compared to losartan [55]. These analogs were the basis for the further development of bis-alkylated derivatives bearing two symmetrically biphenyl tetrazole groups on the two imidazole nitrogens, called bisartans, with notable properties relevant to hypertension and COVID-19 therapy [56,57]. The use of benzimidazole as a scaffold instead of imidazole and bis (biphenyl) tetrazole alkylation resulted in the development of bisartan ACC519TT, which exhibited unique binding affinities due to the tetrazole and increased aromaticity reported in recent articles [58,59]. The interaction of aromatic phenyl groups with arginine, as between ACC519TT and AT_1_R^167^, has been previously reported as a dominant binding factor due to pi-pi electron interactions [60]. The importance of polyfunctional arginine, which has been found to be the target of tetrazole-based drugs, has also been reported as a key amino acid in autoimmune and oncogenic diseases [61,62].

## 5. Conclusions

ARB sartans act as inverse agonists of AT_1_Rs, causing prolonged receptor desensitization. Computer simulations of ARB sartans/bisartans binding to AT_1_Rs, αARs, and µORs indicate that these ligands preferentially bind to the active site on the cell surface of all three GPCR receptors, with a consistent order of ligand preference. Bioassays and docking studies have shown that ARBs block both AngII and alpha receptors, making them potentially more potent drugs than ACE inhibitors. Bioassays confirm that ARBs block αARs and µORs. Sartans/bisartans appear to interfere with the desensitization and/or resensitization (tolerance) mechanisms of αARs and µORs, suggesting their potential role in treating methamphetamine and opioid addiction.

CAD and MD simulations are based on semi-empirical data and theoretical molecular orbital calculations, which are continuously refined and improved. Therefore, the present findings and interpretations are based on evolving concepts and contain hypothetical and speculative elements.

## Data Availability

The datasets used and/or analyzed in this study are publicly available at zenodo.org (https://zenodo.org/records/14504829; accessed 17 December 20204). Software used in the manuscript for analysis of data are third party software packages that can be found at https://www.yasara.org/ (accessed 10 December 20204) and https://www.zbh.uni-hamburg.de/ (accessed 10 December 20204).

## Supplementary Materials

The following supporting information can be downloaded at: https://www.mdpi.com/article/10.3390/biom15060855/s1, Figure S1: Chemical structures of commercially available sartans, experimental sartans and opioids.

## Abbreviations

The following abbreviations are used in this manuscript:

ACC519TT	benzimidazole-bis-N,N′-biphenyl tetrazole
ACC519	benzimidazole-bis-N-biphenyl tetrazole
ACE	Angiotensin-converting enzyme
Ala	Alanine
AngII	Angiotensin II
ARB	Angiotensin receptor blocker
Arg	Arginine
Asp	Aspartic acid
AT1R	Angiotensin II type I receptor
Azil	Azilsartan
A-mode	Resting state
A*-state	Activated state
BisAdilAm	Bisartan-A-diamine
BisAdilAmAc	Bisartan-A-diamino acid
BisACN	Bisartan-A-nitrile
BisCCN	Bisartan-C-nitrile
BisDCN	Bisartan-D-nitrile
BnzCN13	BenzimidazoleCN_13
CAD	Computer-aided docking
Cand	Candesartan
cpd3	CarboxyBenzlmidaoleBisTetrazole_3
cpd4	Nalkylimidazole_4
cpd5	NalkylimidazoleMethylester_5
cpd7	N_Np-alkylimidazole-methyl_7
cpd10	ImidazoleBisTetrazole_10
cpd11	ImidazoleBisCN_11
cpd12	ImidazoleBisCNmetjyl_12
cpd14	BenzlmidazoleCN_COOCH3_14
Cyclaz	Cyclazosin
Cys	Cysteine
DIZE	Diminazene aceturate
Doxaz	Doxazosin
D-state	Desensitized state
Epro	Eprosartan
Gln	Glutamine
Glu	Glutamic acid
Gly	Glycine
GPCR	G-protein couple receptor
His	Histidine
Ibre	Irbesartan
Ile	Isoleucine
Kd	Binding constant
Leu	Leucine
Lisino	Lisinopril
Lo	Losartan
MD	Molecular dynamics
Met	Methionine
Methamp	Methamphetamine
NB	Nanobody
Norepi	Norepinephrine
Olme	Olmesartan
Oxycod	Oxycodone
PASSer	Protein Allosteric Sites Server
PDB	Protein Docking Bank
Phe	Phenylalanine
Pheneph	Phenylephrine
Pro	Proline
RMSD	Root-mean-square deviation
RSV	Respiratory syncytial virus
SARS-CoV-2	Severe acute respiratory syndrome coronavirus 2
Trimaz	Trimazosin
Trp	Tryptophan
Tyr	Tyrosine
Val	Valsartan
α1BAR	Alpha 1B adrenergic receptor
α2CAR	Alpha 2C adrenergic receptor
µOR	μ-opioid receptors
ժOR	ժ-opioid receptors
Å	Angstroms
