# Supplementary material for: Docking Simulations of G-Protein Coupled Receptors Uncover Crossover Binding Patterns of Diverse Ligands to Angiotensin, Alpha-Adrenergic and Opioid Receptors: Implications for Cardiovascular Disease and Addiction
Source: Biomolecules. 2025 Jun 11;15(6):855. doi: 10.3390/biom15060855 (PMC12190924; doi:10.3390/biom15060855)
Supplement: Supplementary file 1 [file biomolecules-15-00855-s001.zip › biomolecules-3588335-supplementary.pdf]

SUPPLEMENTARY FIGURE

Docking simulations of G-protein coupled receptors uncover crossover binding patterns of diverse ligands to angiotensin, alpha-adrenergic and opioid receptors: Implications for addiction

Harry Ridway[1,2], Graham Moore[3,4], John M. Matsoukas[4,5,6,7 *], Laura Kate Gadanec[5*]

[1] Institute for Sustainable Industries and Liveable Cities, Victoria University, Melbourne, VIC 8001, Australia; ridgway@vtc.net (H.R.)
[2] THERAmolecular, LLC, Rodeo, New Mexico, NM 88056, USA
[3] Pepmetics Inc., 772 Murphy Place, Victoria, BC V8Y 3H4, Canada; mooregj@shaw.ca (G.J.M.)
[4] Department of Physiology and Pharmacology, Cumming School of Medicine, University of Calgary, Calgary, AB T2N 1N4, Canada; imats1953@gmail.com (J.M.M.)
[5] Institute for Health and Sport, Immunology and Translational Research, Victoria University, Melbourne VIC 3030, Australia; laura.gadanec@live.vu.edu.au (L.K.G.)
[6] NewDrug/NeoFar PC, Patras Science Park, Patras 26504, Greece
[7] Department of Chemistry, University of Patras, Patras 26504, Greece

* Correspondence: imats1953@gmail.com and laura.gadanec@live.vu.edu

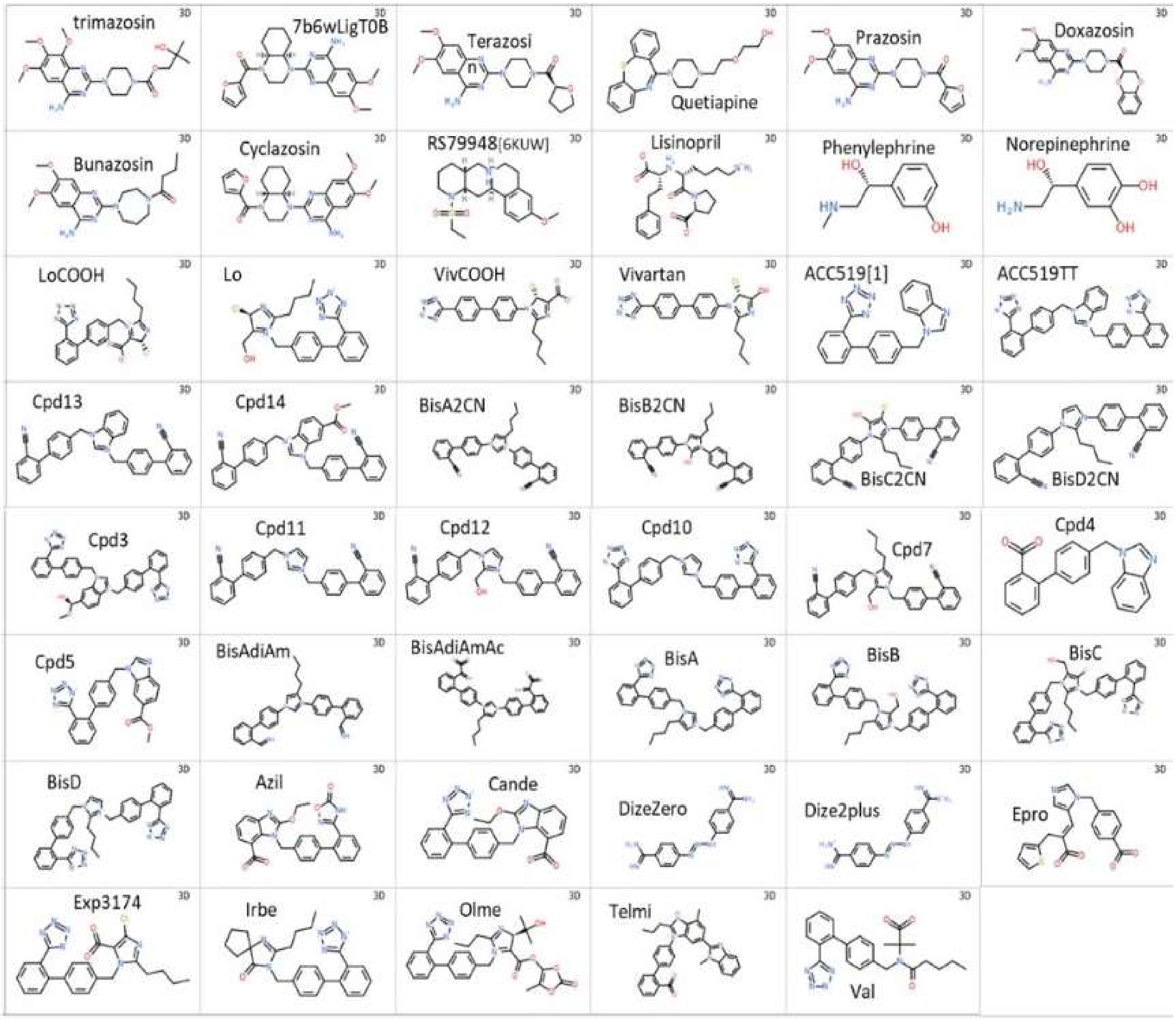

Supplementary Figure S1: Chemical structures of commercially available sartans, experimental sartans and opioids.
